# Precipitation Variability Affects Aboveground Biomass Directly and Indirectly *via* Plant Functional Traits in the Desert Steppe of Inner Mongolia, Northern China

**DOI:** 10.3389/fpls.2021.674527

**Published:** 2021-08-11

**Authors:** Huan Cheng, Yuanbo Gong, Xiaoan Zuo

**Affiliations:** ^1^College of Forestry, Sichuan Agricultural University, Chengdu, China; ^2^Department of Biology, University of Maryland, College Park, MD, United States; ^3^Urat Desert-Grassland Research Station, Northwest Institute of Eco-Environment and Resources, Chinese Academy of Science, Lanzhou, China

**Keywords:** precipitation, plant functional traits, aboveground biomass, ecosystem function, desert steppe

## Abstract

Clarifying the response of community and dominance species to climate change is crucial for disentangling the mechanism of the ecosystem evolution and predicting the prospective dynamics of communities under the global climate scenario. We examined how precipitation changes affect community structure and aboveground biomass (AGB) according to manipulated precipitation experiments in the desert steppe of Inner Mongolia, China. Bayesian model and structural equation models (SEM) were used to test variation and causal relationship among precipitation, plant diversity, functional attributes, and AGB. The results showed that the responses of species richness, evenness, and plant community weighted means traits to precipitation changes in amount and year were significant. The SEM demonstrated that precipitation change in amount and year has a direct effect on richness, evenness, and community-weighted mean (CWM) for height, leaf area (LA), specific leaf area (SLA), leaf dry matter content (LDMC), leaf nitrogen content (LNC), and leaf carbon content (LCC) and AGB; there into CWM for height and LDMC had a direct positive effect on AGB; LA had a direct negative effect on AGB. Three dominant species showed diverse adaptation and resource utilization strategies in response to precipitation changes. *A. polyrhizum* showed an increase in height under the precipitation treatments that promoted AGB, whereas the AGB of *P. harmala* and *S. glareosa* was boosted through alterations in height and LA. Our results highlight the asynchronism of variation in community composition and structure, leaf functional traits in precipitation-AGB relationship. We proposed that altered AGB resulted from the direct and indirect effects of plant functional traits (plant height, LA, LDMC) rather than species diversity, plant functional traits are likely candidate traits, given that they are mechanistically linked to precipitation changes and affected aboveground biomass in a desert steppe.

## Introduction

For the past decades, studies on the relationship between climate change and ecosystem attributes, and potential feedback of plants have sprung up (Bai et al., 2004; Griffin Nolan et al., 2018; Zhang B. et al., 2020) because of growing unexpected climate changes and ecosystem responses. The climatic model predicted that precipitation was likely to be more uncertain (Power et al., 2013) and accompanying an increase in precipitation amount in the future in East Asia (Knapp et al., 2008; Chen and Sun, 2013). Beyond that, researchers also confirmed that precipitation has experienced intense changes in intensity and variability since the last century (Alexander et al., 2006; Trugman et al., 2018; Paschalis et al., 2020). Water availability fluctuations induced by changes in precipitation modulate plant community dynamic and ecosystem function (Yang et al., 2011; Wu et al., 2016; Peralta et al., 2019). For example, changes in the distribution of rainfall events influence the patterns of species richness and species composition (Zavaleta et al., 2003; Báez et al., 2013; Cleland et al., 2013; Libalah et al., 2020), ecosystem net primary productivity (Fay et al., 2003; Heisler-White et al., 2009), and C cycling (Harper et al., 2005). The response of a plant to variation in environment can differ among species, communities, and ecosystems, while a mechanistic comprehension of this modifiability remains open to question.

One helpful avenue to progress our understanding of plant responses to environmental change is the traits-based approach, which pays attention to environmental gradients, plant functional traits across numerous species, and physiologically and morphologically derived common performance (Nicotra et al., 2010; Wellstein et al., 2011). Studies on global patterns in plant height and seed mass are the representative practice (Moles et al., 2007, 2009). Additionally, this approach helps provide a better perspective of understanding on how environmental changes will affect the biosphere in a broad variety of circumstances, including regional climate patterns, biogeochemical cycles, ecosystem services, and functions. For example, plant height and leaf traits (specific leaf area, SLA; leaf nitrogen content, LNC; and leaf area index, LA) have been used to illustrate ecosystem function from tundra (Hudson et al., 2011; Bjorkman et al., 2018), grassland (Zirbel et al., 2017; Xu et al., 2018) to forest ecosystems (Báez and Homeier, 2018; Wang and Ali, 2021). Finegan et al. (2015) reported that biomass-weighted community mean value of max height [community-weighted mean (CWM) H max] was the most important predictor of initial standing biomass, and CWM SLA was the most important predictor of the biomass increment. Finally, they proposed CWM functional traits were strong drivers of ecosystem biomass and carbon-cycle processes in three rainforests. Therefore, the traits-based approach may support uncovering the underlying mechanism of ecosystem response to variation in water availability.

Numerous studies have indicated that the response of community and ecosystem to condition changes depends on the attributes of key species (Huston, 1997; Grime, 1998), relating mass-ratio hypothesis (Suding et al., 2008; Gross et al., 2017). However, the specific role of species in community dynamic remains elusive. Due to complementarity and the selection-effects hypothesis, plant responses to condition changes are not necessarily consistent with coexisting species (Schmid and Harper, 1985; Sinclair and Byrom, 2006). For instance, Zhang R. et al. (2020) found that different from *Stipa glareosa*, dominance of *Allium polyrhizum* and *Peganum harmala*, two of three dominant species, increased in drought treatments. Mahaut et al. (2020) also showed that positive biodiversity influenced both aboveground biomass and a positive complementarity effect resulting from the presence of *Plantago lanceolata* and the CWM trait; on the contrary, the presence of *Sanguisorba* negatively affected productivity in the grassland diversity–productivity relationship test. These results indicated that coexisting species contributed to the ecosystem functioning differently. Thus, disentangling the role of these key species in community functioning will help to develop a deeper comprehension of the adaptation strategies of plants and ecosystem processes.

Under global climate change scenarios, studies investigating on the response of community dynamics and the ecosystem process to climate change are increasing, few studies have investigated desert steppe ecosystems functioning along environmental gradient (Zuo et al., 2020). Desert steppe ecosystems, characterized by water limitations, are thought to be sensitive to climate change, especially precipitation fluctuation (Liu et al., 2016). Xerophytic species provide habitat for desert animals and adjust their attributes to adapt and resist environmental stress (Thomey et al., 2011; El-Keblawy et al., 2015), contributing to maintaining ecosystem productivity (John et al., 2018). However, with the intensification of global climate change and human activities over the last century, desert steppe ecosystem has been suffering degradation and desertification (Mu et al., 2013) because of species diversity loss, imbalance of the xerophytic community structure, and ecosystem function decline. For this reason, more researches on the response of desert steppe ecosystem to changes in precipitation are needed, which may be conducive to restoring fragile ecosystems and to predicting the tendency of the ecosystem dynamic more precisely.

In this study, we aimed to determine the effects of precipitation changes on patterns of ecosystem aboveground biomass by the controlled, manipulated precipitation experiments in a desert steppe. Specifically, we ask three main research questions: (1) Are community composition, species diversity, plant functional traits synchronously affected by precipitation changes in desert steppe? (2) How do the direct and indirect effects of species diversity and functional traits affect community biomass; and (3) what is the role of dominant species in the community responses, relating plant-adaptive and resource-use strategies? Accordingly, we hypothesize that the expected that plant diversity, and functional traits' response to precipitation change alters AGB, and varies with different species.

## Methods

### Study Site

**Figure 1:**
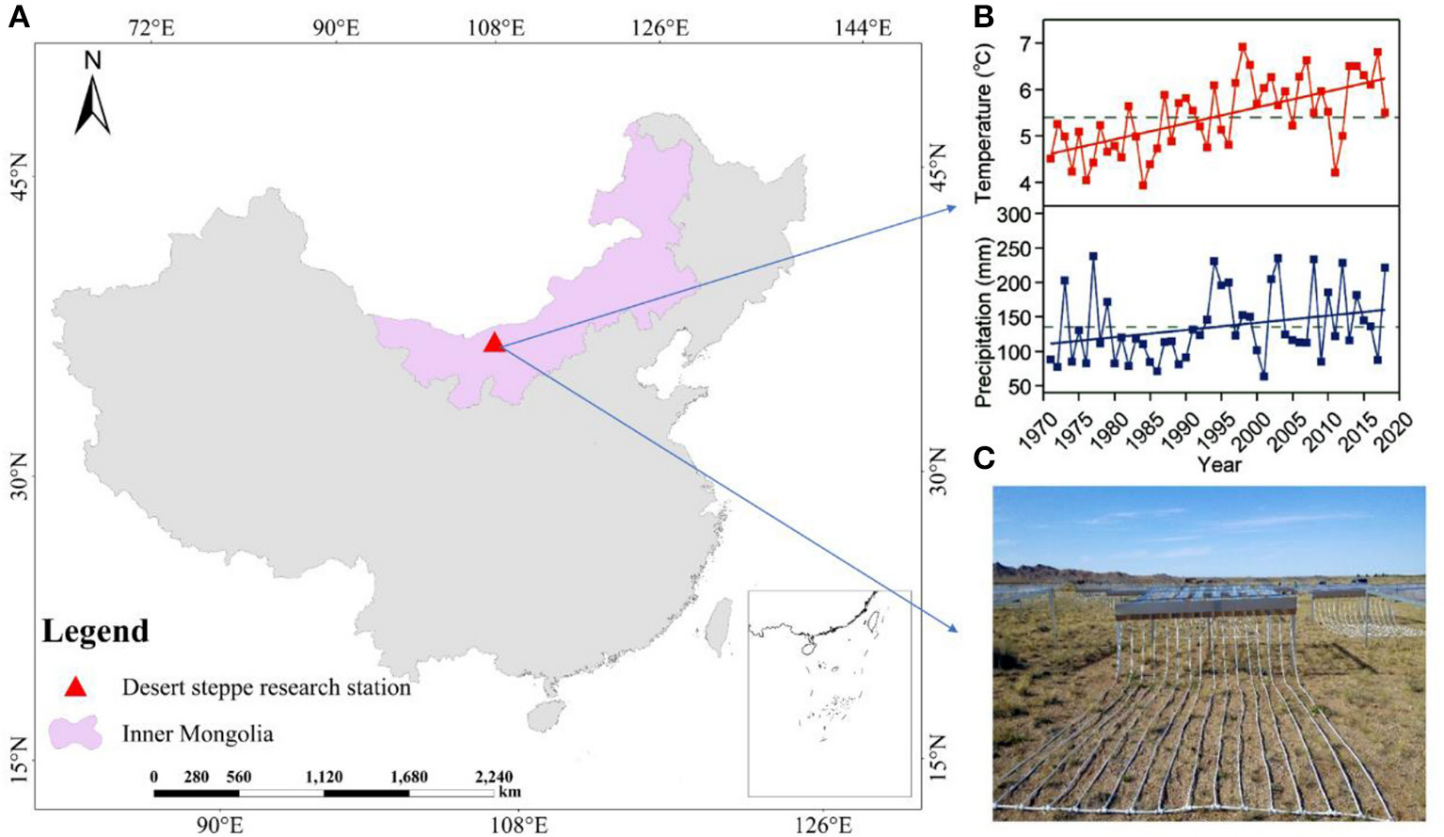
Geographic and climatic characteristics of the study area. **(A)** The map of China depicting the locations of the Urat Desert Steppe Research Station in Inner Mongolia. **(B)** Annual temperature and precipitation from 1971 to 2018. Dotted line represents the mean temperature and precipitation of the last 48 years. The annual mean temperature was 5.4°C. The minimum and maximum temperature in this region were 3.9 and 6.1°C. For the total annual precipitation between these years, the minimum, maximum, and average ones were 63.2, 237.8, and 135.5 mm, respectively. **(C)** Precipitation change treatments were established in 2015 in a desert steppe in Inner Mongolia, China.

The study was conducted at the Urat Desert-grassland Research Station (Northwest Institute of Eco-Environment and Resources, Chinese Academy of Sciences, Figure 1), located in western Inner Mongolia (Zhang R. et al., 2020; Zuo et al., 2020). The site is characterized by brown desert soil and gray-brown desert soil derived from proluvial gravel materials (China Soil Database, Institute of Soil Science, Chinese Academy of Sciences, 2019, http://vdb3.soil.csdb.cn/). Climatic conditions of this study area were measured continuously and recorded by the Hailisu National Meteorological weather station. The study area has a typical temperate continental monsoon climate, affected by the Mongolian continental high-pressure air mass and characterized by a mean annual temperature of 6.3°C (1971–2018) and a mean annual precipitation of 140 mm (1971–2018; Figure 1B). The vegetation is dominated by *Stipa glareosa* P. Smirn., *Peganum harmala* L., and *Allium polyrhizum* Turcz. ex Regel. And *Allium mongolicum, Artemisia frigida* Willd., *Convolvulus ammannii* Desr., and *Salsola collina* Pall were found in our study area, but their contribution to total biomass is limited.

### Experimental Design

**Figure 2:**
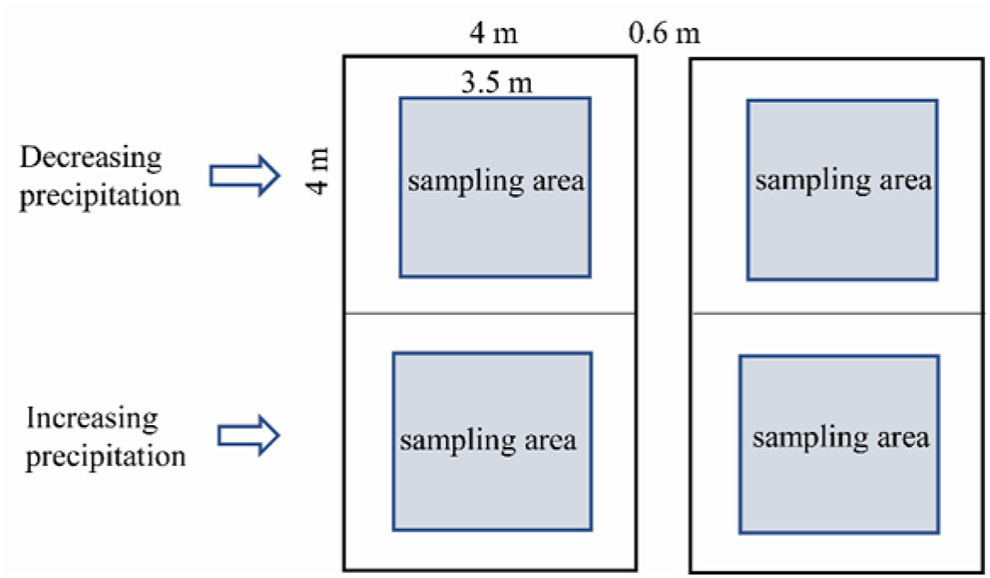
Experimental design.

The experimental design was implemented at the Urat Desert-Grassland Research Station (Figure 1A). In June, 2015, a manipulative, precipitation decrease and increase experiment was established using V-groove collector and drip irrigation distributor (Figure 1C). The rainout shelter (decreased precipitation systems) was 1.5 m high, and the V-groove collector was set on stainless-steel support with a 15° inclination. Polycarbonate plastic V-groove collector (nearly 90% penetration of UV radiation) were mounted in stainless-steel structure to collect water to induce precipitation by −60, −40, and −20%. A drip irrigation distributor consisting of stainless-steel containers in front of the V-grooves was connected to PVC pipes with holes to evenly distribute water that increased precipitation by +60, +40, and +20% (Figure 1C). The V-groove collector and drip irrigation distributor separately covered 16 m^2^ (4 × 4 m) and were 0.6 m apart. The experiment was randomized complete block design, with six replications for each treatment (Figure 2). The control plots without shelters received natural precipitation. In this manner, we manipulated precipitation with 60% reduction to 60% increment, respectively, relative to the natural precipitation. All the treatments were applied from May 2015, 2 years before measurements. Totally, there were 42 sampling plots, including 6 for treatments and 1 for control. These precipitation levels cover the recorded long-term variability in local precipitation, and forming a precipitation gradient, ranging from extreme drought to high precipitation (Figure 1B).

### Measurement of Precipitation Amount

Precipitation data were obtained from the weather station of Urat Desert-grassland Research Station (Figure 1B). The natural growing season precipitation (from May to September) were 28.8 and 21.4 mm in 2017 and 2018. In 2017, actual precipitations under −60, −40, −20, +20, +40, and +60% treatments during the growing season were 11.52, 17.82, 23.04, 28.8, 34.56, 40.32, 46.08 mm, respectively. In 2018, actual precipitations under −60, −40, −20, +20, +40, and +60% treatments during the growing season were 8.65, 12.84, 17.12, 21.4, 25.68, 29.96, 34.24 mm, respectively.

### Community Composition and Plant Diversity

The manipulated experiment was constructed in 2013, and the sampling of this study was carried out in August 2017 and 2018. In 42 plots, plant coverage and height of each species were measured, and the number of individuals was counted.

Based on the collection data of plant coverage, height and numbers, the Shannon-Wiener diversity index and Pielou evenness index were calculated:

Shannon-Wiener diversity index (H):

$$\begin{array}{l} H\;=\;-\;\overset{S}{\underset{i=1}{\sum }}\;\left(N_{i}\;ln\;N_{i}\right) \end{array}$$

Pielou evenness index (J):

$$\begin{array}{l} J\;=\;H/\ln\;S \end{array}$$

where Ni is the relative abundance of species i and S is the total number of individuals.

### Plant Functional Trait

Plant samples were collected at each site for functional trait measurements in mid-August 2017 and 2018. During the current growing season, several fully matured and healthy leaves were collected from 5 to 10 individuals of the dominant species within each plot to measure leaf functional traits according to standard methodologies (Wu et al., 2016). These traits including plant height, leaf area, specific leaf area (SLA), leaf dry matter content (LDMC), leaf thickness, leaf carbon content (LCC), and leaf nitrogen content (LNC) (Cornelissen et al., 2003), which were determined by an elemental analyzer (Costech ECS 4010, Italy). CWM summarized traits variation data as determined mainly by the dominant species (Valencia et al., 2015).

### Response of Species Diversity and Aboveground Biomass to Precipitation Changes

To determine the effect of precipitation changes on community composition, a general linear model was performed with precipitation as an independent variable and community richness, Shannon's diversity index, and Pielou's evenness index as dependent variables. Goodness of fit for regressions was used by comparing their *R*^2^values. The effects of precipitation changes were significant when *p* < 0.05, and analyses were conducted, using the function “lm.”

### Effect of Precipitation and Year on Functional Traits of Community-Weighted Means and Dominant Species

We calculated the CWM trait value for each plot:

(1) $$\begin{array}{l} \text{CWM trait =}\;\sum {\text{p}}_{\text{i}}{\text{x}}_{\text{i}} \end{array}$$

where CWM trait is the CWM for the x trait and p_*i*_ and x_*i*_ are the relative coverage and the trait value of species i in the community. CWM represents the community-level trait value and is controlled by the trait values of the dominant species weighted by their coverage for each plant functional trait on the 42 plots separately, using the “dbFD” function in the R package “FD.”

We used ANOVA (with pairwise Tukey tests) to compare the CWMs trait distributions in the six precipitation treatments.

### Response of CWM Traits

We built linear models of plant functional traits using a Bayesian approach, ranging in complexity from a single term to a two-way interaction with a focus on addressing the question of whether year, precipitation, or year-and-precipitation interactions influence plant functional traits. Thus, three models were run for each CWM trait. In all models, the CWM trait followed a normal distribution:

Plant functional trait_i_ ~ dnorm (mu_i_, sigma_i_)

where mu_i_ is the trait of each plot or species, sigma_i_ is the variance, and i is each plot or species. linear models were fitted where the plant functional trait was a function of precipitation, year, or precipitation and year interactions, where mu_i_ was the plant functional trait of each plot or species, α was the model intercept, b1 was the coefficient of precipitation, b2 was the coefficient of a year, and b3 was the coefficient of precipitation and year interactions.

$$\begin{array}{l} mu_{i}=a\;+\;b1\;*\;precipitation\\ mu_{i}=\;a\;+\;b1\;*\;precipitation\;+\;b2\;*\;year\\ mu_{i}=a\;+\;b1\;*\;precipitation\;+\;b2\;*\;year\\ \;+\;b3\;*\;precipitation\;*\;year \end{array}$$

### Response of the Dominant Species Traits

The role of species is rarely considered in models that assess the impact of functional traits. We built linear mixed-effects models of growth, using a Bayesian approach, ranging complexity from a single term to having a two-way interaction, with a focus on addressing whether species, year, precipitation, or year-and-precipitation interactions influenced leaf functional traits.

$$\begin{array}{l} mu_{j}=\;a_{j}\left[spp\right]\;+\;b_{j}\left[spp\right]\;*\;precipitation\\ mu_{j}=\;a_{j}\left[spp\right]\;+\;b_{j}\left[spp\right]\;*\;precipitation\;+\;d_{j}\left[spp\right]\;*\;year\\ mu_{j}=\;a_{j}\left[spp\right]\;+\;b_{j}\left[spp\right]\;*\;precipitation\;+\;d_{j}\;\left[spp\right]\;*\;year\\ \;+z_{j}\left[spp\right]\;*\;precipitation\;*\;year \end{array}$$

where mu_j_ was the leaf functional trait of each species, α_j_ was the model intercept, [spp] was the random effects of species, b_j_ was the coefficient of precipitation, d_j_ was the coefficient of year, and z_j_ was the coefficient of the precipitation-and-year interaction.

In all models, the CWM trait (mui) and functional trait (muj) were modeled as normally distributed, and the variance hyperparameters were given diffuse gamma priors: *N* (mean = 0, precision = 0.01). We used the Wantanabe–Akaike information criterion (WAIC). The WAIC is a fully Bayesian information criterion valid for hierarchical models (Hooten and Hobbs, 2015). As with the other model selection criteria, smaller value of WAIC indicates a greater model predictive ability. All analyses were performed in R statistical software, version 3.6.1 (R Development Core Team, 2019).

### Causal Relationship of Precipitation, Species Diversity, Plant Functional Traits, and AGB

Structural equation models were employed to analyze the causal relationship of precipitation variables, plant diversity variables, and functional trait, and AGB (a total of 12 variables). To develop the final SEMs, we started with our initial hypothesized relationships among the variables. A Pearson correlation analysis was conducted on plant diversity, precipitation, and functional traits (Supplementary Tables 6, 7). The decision to remove a path was based on the performance of the overall model fit and the *p*-value for the path. To simplify the SEMs, we first deleted the functional traits with no significant relationship with precipitation and year according to the results of the previous correlation analysis. We did not establish the relationships between functional traits. In addition, we hypothesized that climatic variables would significantly affect plant functional traits in the optimal model. We deleted the correlation between precipitation, year, and plant functional traits when precipitation and year did not significantly affect plant functional traits or if their addition led to a decrease in the best model interpretation. Model evaluation was determined by the chi-square (χ^2^) test (*p* > 0.05 for a satisfactory fit) and the standardized root mean square residual (SRMR < 0.05 for a satisfactory fit). The Akaike information criterion (AIC) was used to select the best model with a satisfactory fit. When a model met the criteria of the chi-square test and SRMR but contained non-significant paths in the relationship between precipitation, plant diversity indexes, and functional traits, we repeated the model fitting and evaluation by removing these paths. The total standardized effect that one variable had on another equaled the sum of its standardized direct and indirect effects. Non-significant paths of the relationship between precipitation, plant diversity indexes, and functional traits were not shown. Then we used the same criteria to structure the SEM model to show the causal relationship of precipitation, plant functional traits, and AGB of the dominant species level using the Amos graphics software.

## Results

### Patterns of Species Composition and Coverage Under Manipulated Precipitation Treatments

**Figure 3:**
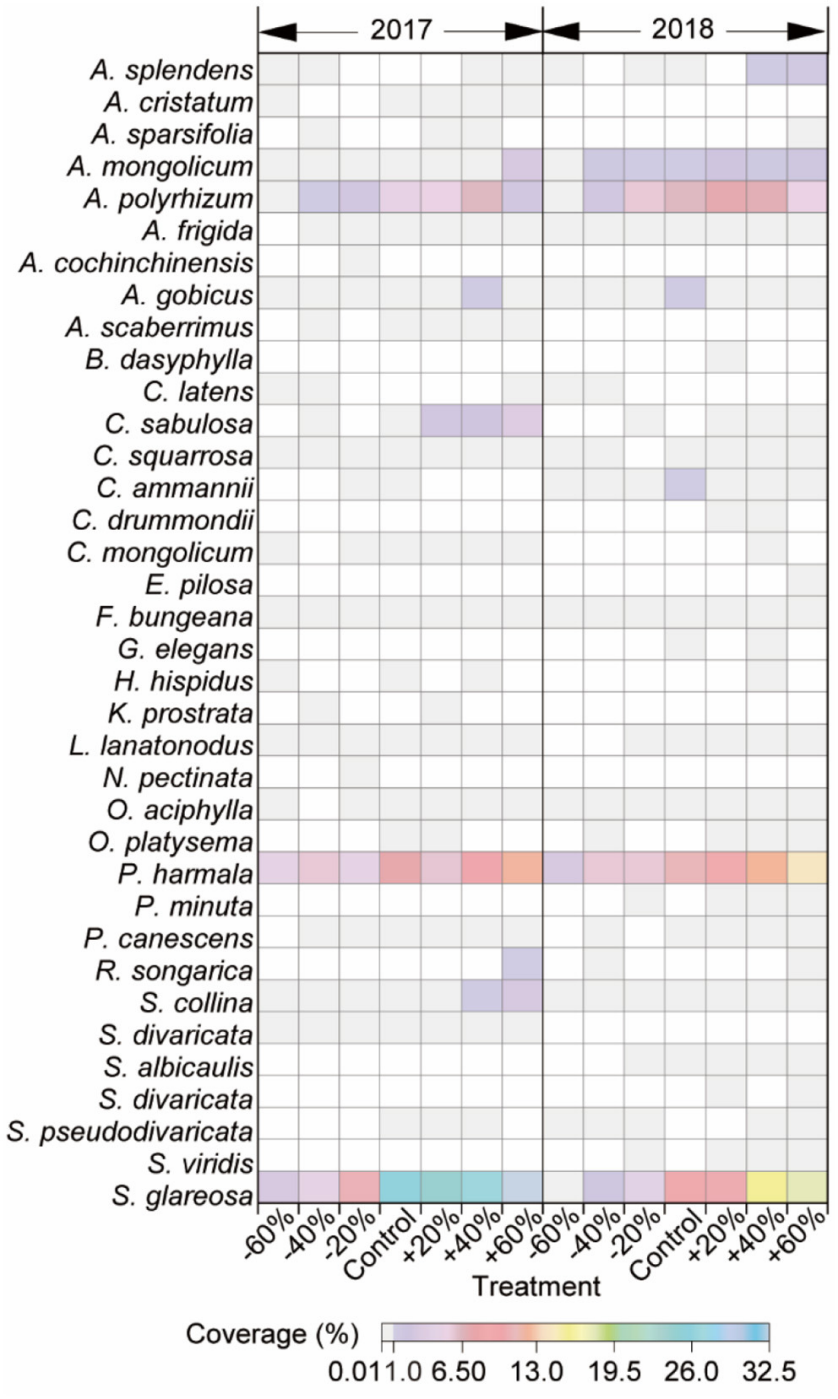
Abundance of 36 species in this study under −60, −40, −20, +20, +40, +60% precipitation treatment, and control in 2017 and 2018. The color of each panel is proportional to the value of abundance.

About 14 families, 14 genera, and 36 species were collected in this study, and most were perennials. *A. polyrhizum, P. harmala*, and *S. glareosa*, with higher coverage relative to others, responded dramatically to precipitation variation and were defined as dominant species (Figure 3, Supplementary Table 5). Increasing and decreasing precipitation affected species composition. Concretely, *Neopallasia pectinata, Kochia prostrata, Astragalus scaberrimus, Agropyron cristatum*, and *Asparagus cochinchinensis* were present in 2017, and *A. cochinchinensis* and *N. pectinata* were only in plots receiving −20% precipitation alteration. While *Setaria viridis, Saposhnikovia divaricata, Scorzonera albicaulis, Plantago minuta, Gypsophila elegan., Coreopsis drummondii*, and *Bassia dasyphylla* were present in 2018, and *Eragrostis pilosa, Bassia dasyphylla*, and *S. divaricata* were only in plots receiving +20, +40, or +60% precipitation alterations (Figure 3).

### Sensitivity of Dominant Species to Precipitation Change

**Figure 4:**
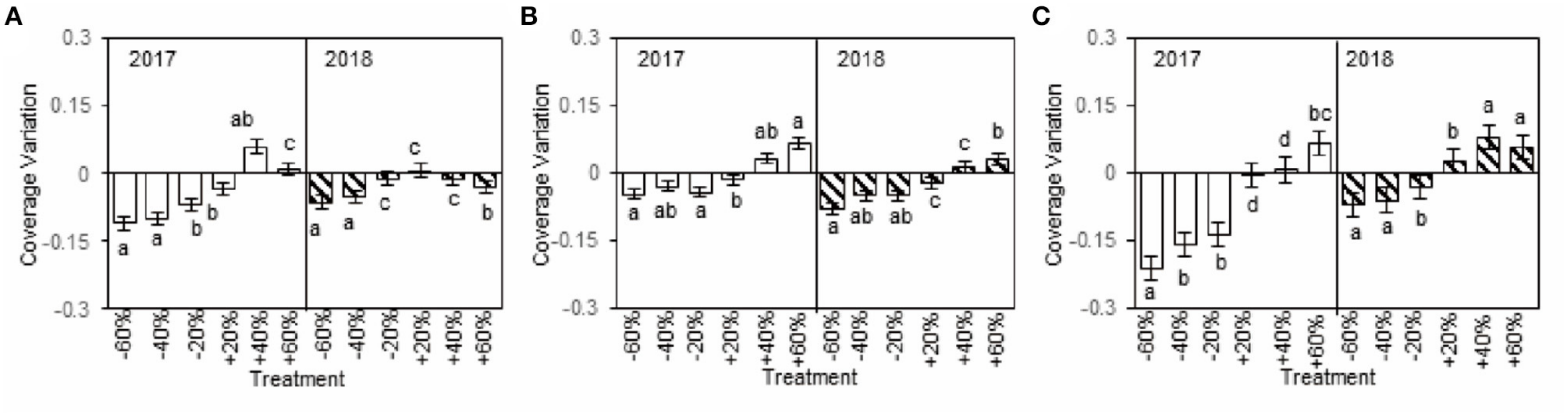
Coverage variations of six precipitation treatments of *A. polyrhizum* **(A)**, *P. harmala* **(B)**, and *S. glareosa* **(C)**, compared to control in 2017 and 2018. The different capital letter indicates significant under difference treatments in 2017 and 2018 at *p* < 0.05 by Duncan test.

The sensitivity of dominant species to precipitation changes was reflected by coverage variation in our study. The coverage variations of *A. polyrhizum, P. harmala*, and *S. glareosa* were considerable and negative with decreased precipitation, while, under increased precipitation, the variations tended to be positive (+40 and +60% treatments), although this finding was not always the case (+20% treatment, Figure 4), and the coverage variation of the three dominant species varied between 2017 and 2018 (Figures 3, 4). Additionally, the coverage variations of dominant species were related to alterations in precipitation intensity. Specifically, in the plots with decreased precipitation, the absolute coverage variation of *A. polyrhizum* reached a maximum under the −60 and −40% precipitation treatment in 2017, *P. harmala* reached a maximum under the −60% precipitation treatment in 2018, and *S. glareosa* reached a maximum under the −60% precipitation treatment in 2017 (Figure 4). In the increased precipitation treatments, the absolute coverage variation of *A. polyrhizum* reached a maximum under the +40% precipitation treatment in 2017, *P. harmala* reached a maximum under the +60% precipitation treatment in 2017, and *S. glareosa* reached a maximum under the +40% precipitation treatment in 2018 (Figure 4).

### Response of Species Diversity Index and AGB to Precipitation Change

**Figure 5:**
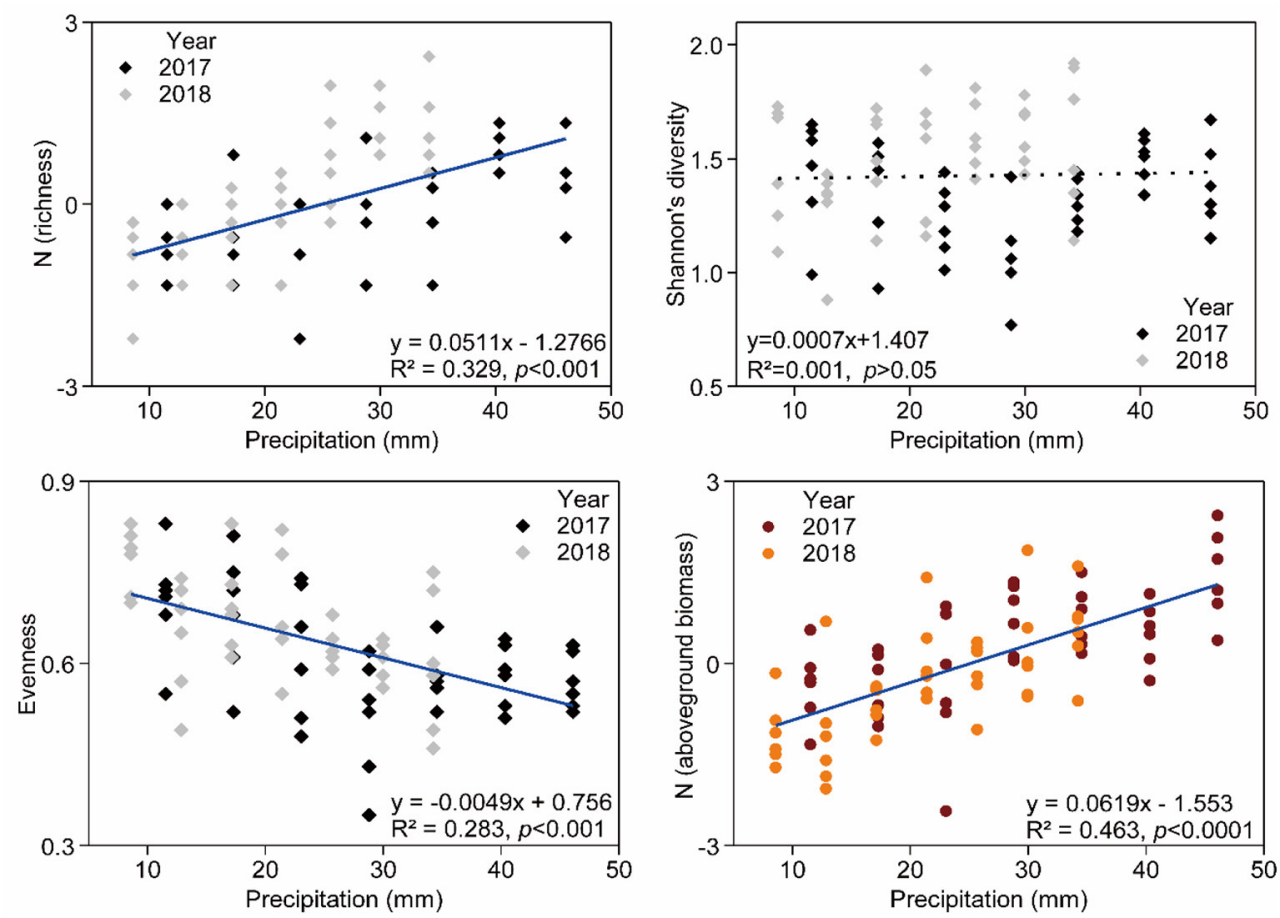
General linear regression analysis for richness, Shannon's diversity, evenness, aboveground biomass, and precipitation amount. Details of the fitted models are given within each panel.

Relationship between species richness, Pielou evenness indexes, community AGB, and precipitation were significant (Figure 5). Species richness (*R*^2^ = 0.329, *p* < 0.001) and community AGB (*R*^2^ = 0.463, *p* < 0.001) were positively correlated with increasing precipitation, and Pielou evenness (*R*^2^ = 0.283, *p* < 0.001) was negatively associated with increasing precipitation. However, there was no significant effect of precipitation change on Shannon's diversity (Figure 5).

### Plant Functional Traits Under Precipitation Treatments

**Figure 6:**
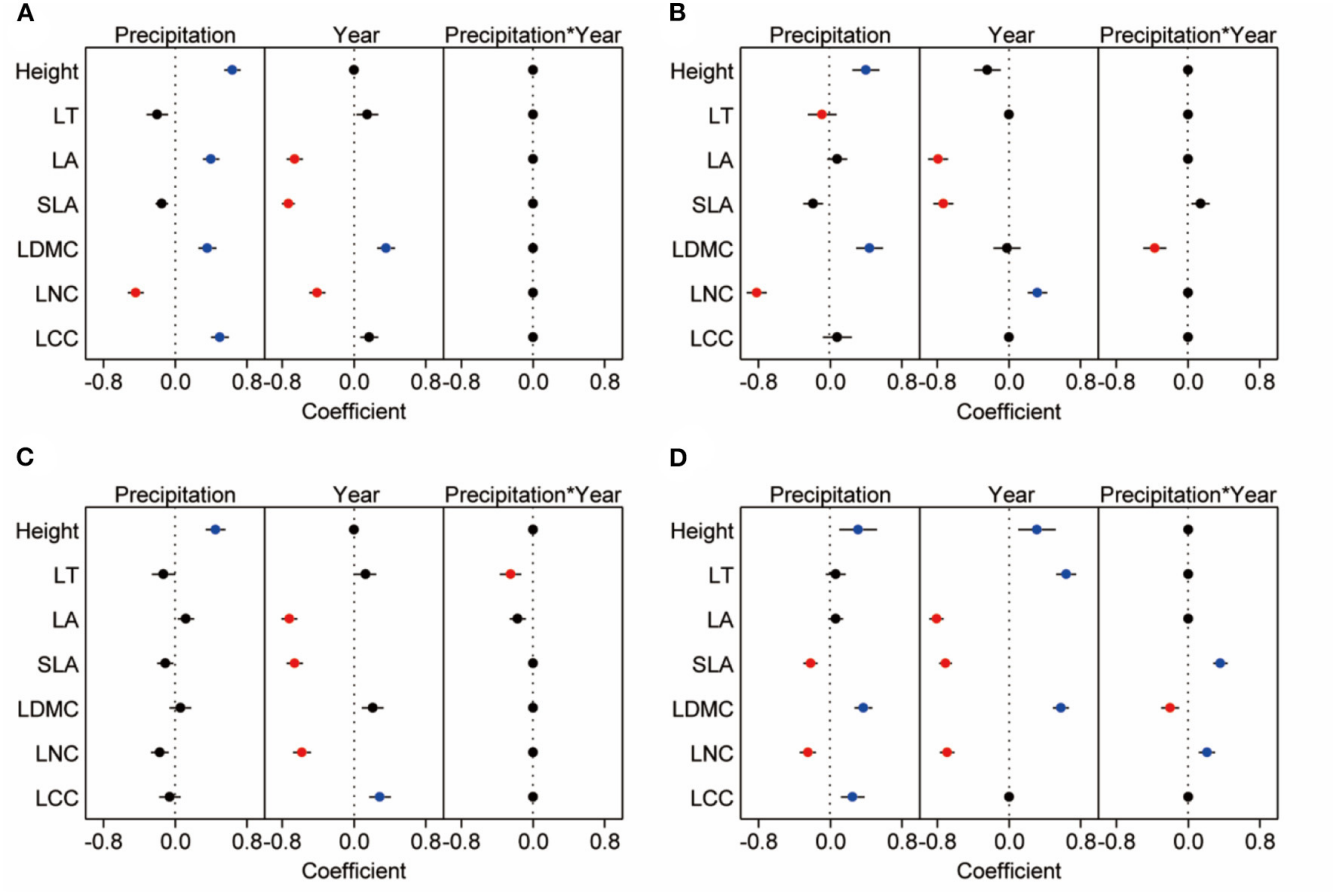
The best model of community-weight means **(A)** and dominant species (*S. glareosa*, **B**; *P. harmala*, **C**; and *A. polyrhizum*, **D**) traits response to precipitation, year and precipitation*year. We extract the best from three possible Bayesian models, each row represents the model of traits, which are influenced by precipitation, year and precipitation*year. Blue dots indicate significant and positive response, red dots indicate significant and negative response, and black dots indicate non-significant or no response.

**Figure 7:**
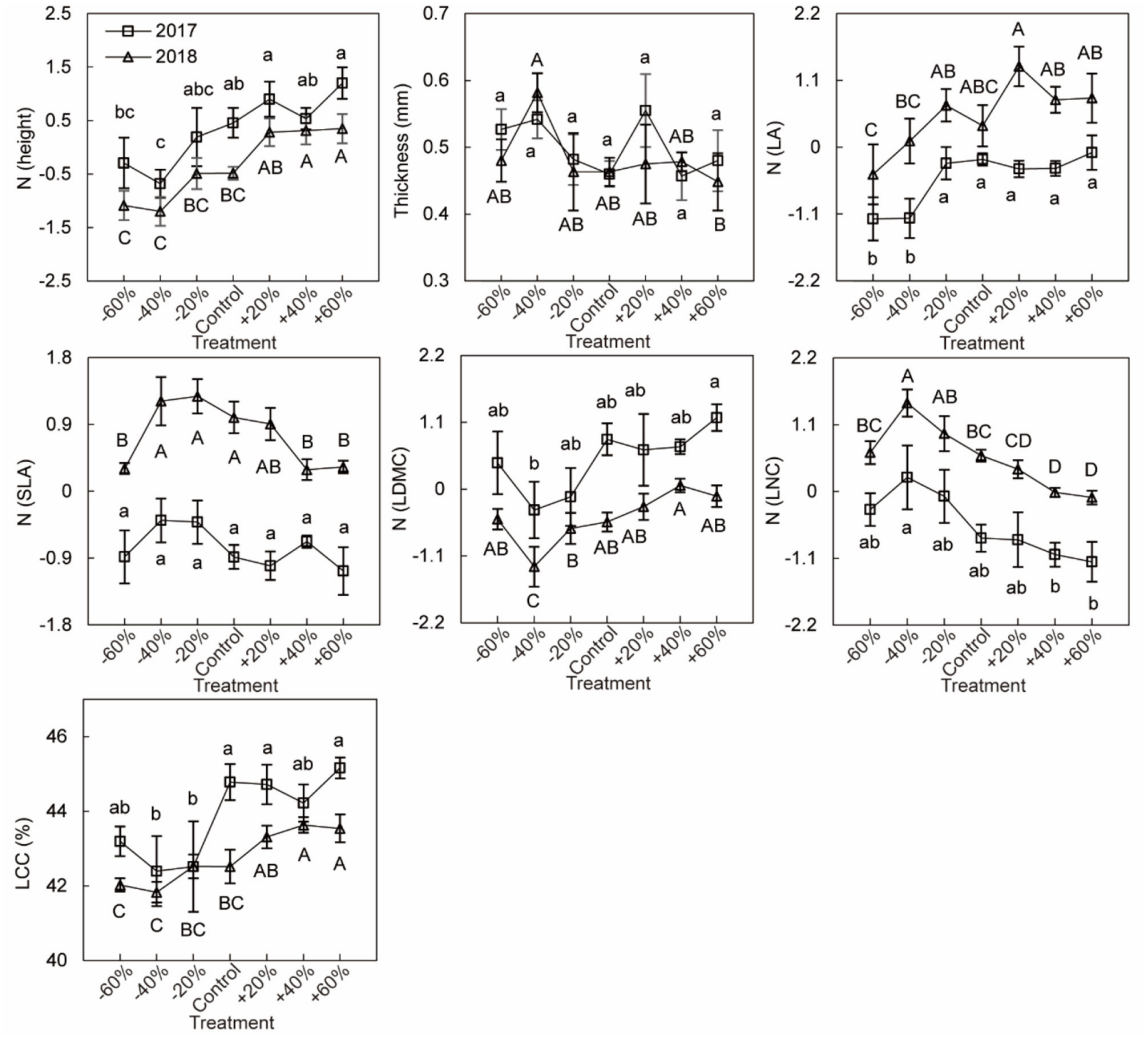
Community-weight mean leaf traits under six precipitation treatments (−60, −40, −20, +20, +40, +60%) and control. Values represent means ± standard errors (*n* = 6). The different lowercase letters indicate significant difference in 2017 at *p* < 0.05 by Duncan test. The different capital letters indicate significant difference in 2018 at *p* < 0.05 by Duncan test.

Based on linear models by Bayesian approach, we found precipitation amount changes had greater effects (with a greater coefficient) than year on CWM for height, LNC, and LCC, while the year had greater effects (with a greater coefficient) than precipitation amount changes on CWM for SLA and LA (Figure 6A). Under different precipitation treatments, significant decrease of the CWM LNC by increasing precipitation was mainly seen in plots receiving +40 and +60% precipitation treatments. And CWMs of height (especially in plots receiving +40 and +60% precipitation treatment), LA, LDMC (especially in plot receiving +60% precipitation treatment), and LCC (+60% treatment) increased with increasing precipitation. The CWMs of LA, SLA, and LNC were higher in 2018 than in 2017, involving all precipitation treatment (Figures 6, 7, Supplementary Tables 1, 2). For three dominant species, effects of precipitation and year on the leaf functional traits of species were species-specific. Response of height, leaf thickness (LT), LDMC, LNC of *A. polyrhizum*, height of *P. harmala*, and height, SLA, LDMC, LNC, and LCC of *S. glareosa* to precipitation were significant. Significant responses of functional traits of three dominant species to year and precipitation and year interaction were mainly shown in LT, LA, SLA, LDMC, LNC (lowest AIC, Figures 6B–D, Supplementary Tables 3, 4, 8).

### Relationships Among Precipitation, Plant Functional Traits and AGB

**Figure 8:**
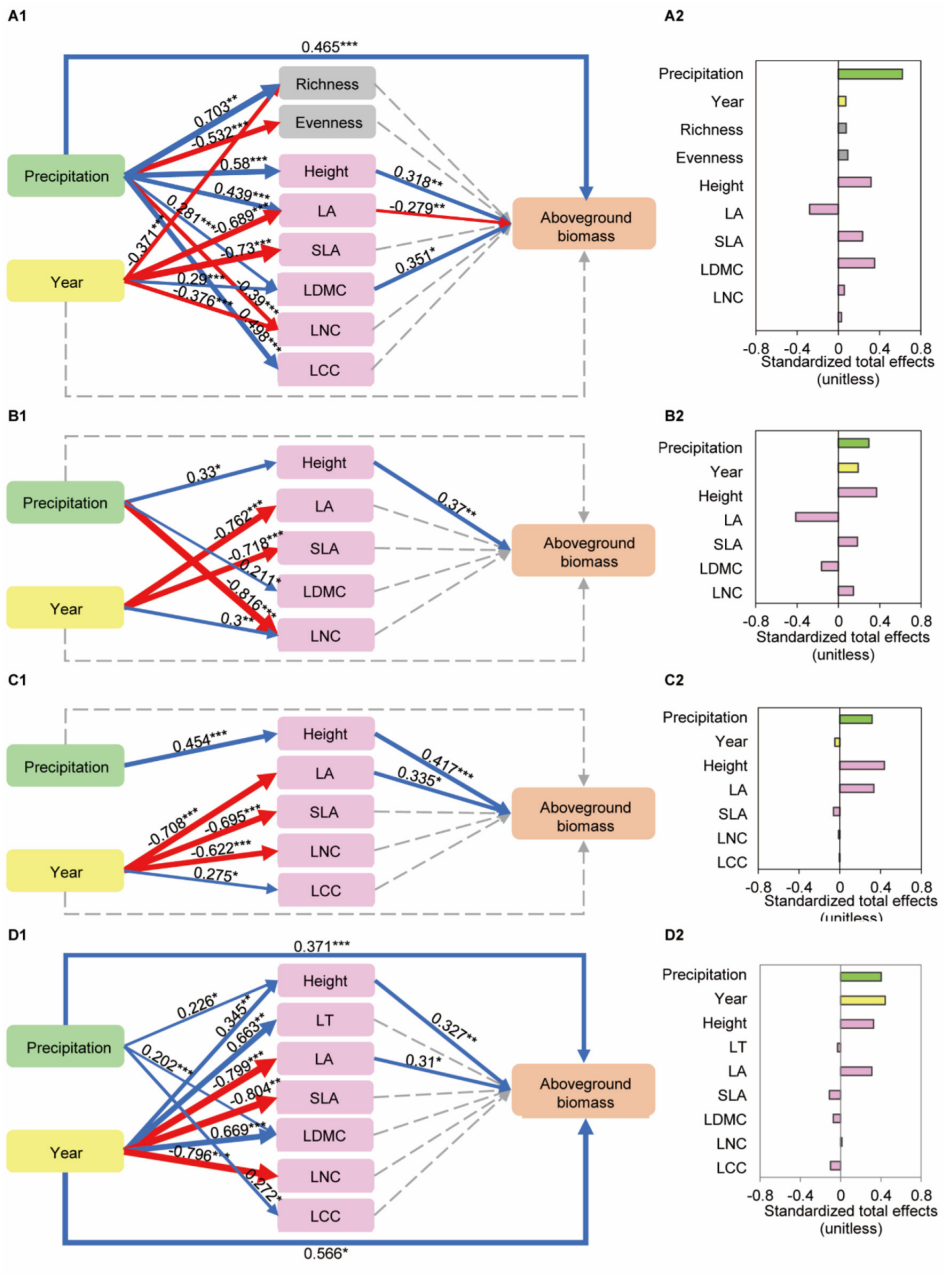
Structural equation models (SEM) of the effects of precipitation and year on aboveground biomass of a community and three dominant species. **(A1)** CWM leaf traits: *p* = 0.102, df = 17, χ^2^ = 24.691, GFI = 0.949, RMSEA = 0.071, AIC = 122.691. **(A2)** Total standardized effects from the SEM of precipitation and year on community aboveground biomass. **(B1)** *A. polyrhizum*: *p* = 0.537, df = 11, χ^2^ = 9.921, GFI = 0.952, RMSEA = 0.000, AIC = 59.921. **(B2)** Total standardized effects from the SEM of precipitation and year on aboveground biomass of *A. polyrhizum*. **(C1)** *P. harmala*: *p* = 0.341, df = 13, χ^2^ = 14.482, GFI = 0.953, RMSEA = 0.04, AIC = 60.482. **(C2)** Total standardized effects from the SEM of precipitation and year on aboveground biomass of *P. harmala*. **(D1)** *S. glareosa*: *p* = 0.481, df = 16, χ^2^ = 15.607, GFI = 0.954, RMSEA = 0.000, AIC = 93.607. **(D2)** Total standardized effects from the SEM of precipitation and year on aboveground biomass of *S. glareosa*. The blue and red arrows represent significant positive and negative pathways, the gray dashed arrows represent no significant pathways, respectively. Numbers adjacent to the arrows are standardized path coefficients, analogous to relative regression weights and indicative of the effect size of the relation. The thickness of the arrows is proportional to the magnitude of the standardized path coefficient s. The arrow width is proportional to the strength of the relationship.

In final SEM (*p* = 0.102, df = 17, χ^2^ = 24.691, GFI = 0.949, RMSEA = 0.071, AIC = 122.691, Figures 8A1,A2, Supplementary Table 9) for predicting the direct and indirect effects of precipitation and year changes on aboveground biomass of community. Precipitation (β = 0.622, a standardized coefficient) had an indirect effect on AGB of a community through CWM of height (0.318), LA (−0.279), LDMC (0.351). Precipitation had positive directive effects on height (0.58), LA (0.439), LDMC (0.281), whereas a year had a negative direct effect on LA (−0.689) and had a positive direct effect on LDMC (0.29). The total variance in AGB explained by these variables was close to 59.5%. These results implied that a variation in height, LA, LDMC due to precipitation and year changes resulted in an increase in AGB.

The AGB of *A. polyrhizum* was, however, driven by height owing to precipitation and year changes (*p* = 0.537, df = 11, χ^2^ = 9.921, GFI = 0.952, RMSEA = 0.000, AIC = 59.921, Figures 8B1,B2, Supplementary Table 10). For *P. harmala* (*p* = 0.341, df = 13, χ^2^ = 14.482, GFI = 0.953, RMSEA = 0.04, AIC = 60.482, Figures 8C1,C2, Supplementary Table 11) and *S. glareosa* (*p* = 0.481, df = 16, χ^2^ = 15.607, GFI = 0.954, RMSEA = 0.000, AIC = 93.607, Figures 8D1,D2, Supplementary Table 12), precipitation and a year had significant effects on AGB through height and LA.

## Discussion

### Precipitation Treatments Changed Community Composition and Species Diversity

We found a positive linear relationship between species richness and precipitation, implying that precipitation change influenced community composition. *E. pilosa* and *A. polyrhizum* were found to be more abundant in plots receiving increased precipitation in our study, which was characterized by rapid growth and low water-use efficiency (Blumenthal et al., 2020). Enhanced water availability contributed to the germination of dormant seeds or vegetation tissue of these species (Finch Savage and Leubner Metzger, 2006), plant photosynthesis and respiration (Huxman et al., 2004), further promoting growth and reproduction (Huang et al., 2018), consequently regulating community composition and species richness (Walck et al., 2011). *A. polyrhizum, P. harmala*, and *S. glareosa*, with higher coverage, responded to precipitation changes dramatically. A remarkable increase in dominant species abundance resulted in the increased dominance of these three species while the dominance of rare species decreased, explaining the negative linear relationship between evenness and precipitation. However, there is no significant effect of precipitation change on Shannon's diversity index, this may relate to condition-type specific (Thuiller et al., 2005; Irl et al., 2015). For example, a study in the Great Plains of USA (Byrne et al., 2017) reported that a positive linear relationship between precipitation and Shannon's diversity in shortgrass steppe and a negative relationship in a mixed grass prairie. In addition, in tropic forests and savannas, negative or unimodal patterns were found in species' or communities' responses to increasing precipitation and the relationship was amplified by biological and abiotic processes (Givnish, 1999). Hence, the effect of climatic factors on species diversity needs to be further explored in different ecosystem types.

### Plant Functional Traits Were Altered by Precipitation Treatments and Year Variation

The present study indicated that CWMs of plant height, LA, LDMC, and LCC increased, and LNC decreased with increasing precipitation, which supported the leaf economics spectrum (Wright et al., 2004), indicating higher acquisition and turnover of resources in plants with increasing water availability (Wright et al., 2004). Plant height is supposed to be a central part of the plant ecological strategy from our study and other previous studies (Westoby, 1998; Falster and Westoby, 2003; Moles et al., 2009; De Frenne et al., 2011). Moles et al. (2009) reported a remarkably tight relationship between latitude and height, pointing out that plant height was strongly correlated with a life span, seed mass, and time to maturity. With an increase in precipitation, the photosynthetic rate was promoted by increasing the leaf area, and more biomass accumulated in a short time (Wright et al., 2001; Barker et al., 2006; Wilcox et al., 2021). Increased precipitation should cause a pronounced shift toward communities with taller plants and with more resource acquisition and storage leaves (high LA, LDMC, and LCC). These relationships underline the potential importance of plant stature for growth, survival, and adaptation, and they may influence ecosystem dynamics and services during climate or condition changes (Lv et al., 2019). Furthermore, LA, SLA, LDMC, and LNC, “growth investment” traits changed greatly by year (Schellenberger Costa et al., 2018), as shown by greater CWMs for “fast” traits during wet years (Poorter and Bongers, 2006; Reich, 2014) and greater values for LDMC related to more tolerance during dry years (Markesteijn et al., 2011).

Additionally, we found that the extent of plant functional trait responses to precipitation changes varies with species type, implying the different adaptive and resource-use strategies of the three species (Reich et al., 2003; Fort et al., 2013). *A. polyrhizum* exhibited significant responses to precipitation changes in six of the seven traits (except for LCC), which was more sensitive to short-term precipitation changes (precipitation treatments) with greater height and LDMC, thinner thickness, and lower LNC to increased precipitation, which is related to a fast-growth strategy and fast resource acquisition (Reich, 2014). *P. harmala* was more tolerant to precipitation changes (Ahmed and Khan, 2010), with five significant traits (except for LT and LDMC). We believe that *S. glareosa* showed greater fitness by regulating more leaf traits (seven traits) when subjected to environmental stress or habitat change, which is related to its slow-growth strategy and slow resource acquisition.

### Precipitation and Year Regulated AGB *via* Plant Functional Traits

Our study explains the pathway of precipitation effect on AGB in dryland. SEM analyses demonstrated that the patterns of CWMs for height, LA, and LDMC altered by the precipitation treatment and a year were strongly related to AGB. These results are in line with those of other studies that showed that CWMs have an important consequence for ecosystem functioning along environment gradient (Diaz et al., 2007; Roscher et al., 2012; Chiang et al., 2016; Cadotte, 2017). Van'T Veen et al. (2020) suggested that temperature and precipitation explained additional 22.1% of the variation in productivity, and that functional trait composition was an important predictor of grassland productivity in Switzerland grasslands. Combining previous studies, we believe that leaf functional traits (except for LT) are good response traits to precipitation change at the community level (Gross et al., 2008; Suding et al., 2008; Sterk et al., 2013). While the altered AGB results from direct effects of precipitation change on the part of response traits (plant height, LA, and LDMC).

An investigation of how traits of dominant species respond to precipitation changes and how they influence AGB can help understand the role of species in ecosystem dynamics and resource-use strategies (Lawton, 1994; Smith and Knapp, 2003; Sinclair and Byrom, 2006). In this study, the response of the plant height of *A. polyrhizum* to short-term precipitation changes affected AGB, supporting Hu et al. (2019). The shallow-root system, large mesophyll cells, and chloroplasts of *A. polyrhizum* promoted the utilization of surface soil water and provided the foundation for the high photosynthetic rate under conditions of sufficient water supply (Ivanov et al., 2004; Hu et al., 2019). Thus, *A. polyrhizum* was defined as an opportunist in our study and was characterized by fast growth in height within a short time, high water content in its leaves, and low water-use efficiency (Blumenthal et al., 2020). The response and adaptation strategies of *P. harmala* and *S. glareosa* were different from those of *A. polyrhizum*. Precipitation and annual changes affected AGB *via* height and LA in *P. harmala* and *S. glareosa*; they developed a great number of cells and chloroplasts in its leaves and a large leaf assimilation surface that provided greater photosynthetic rates and higher efficiency of water use (Ivanov et al., 2004; Baiakhmetov et al., 2020), resulting in a higher capacity for carbon storage and more fitness under water limitation. Overall, *P. harmala* and *S. glareosa* with more fitness and tolerance, are key species for maintaining ecosystem stability in this study (Richmond et al., 2005; Brotherton and Joyce, 2015), helping the ecosystem withstand disturbance, such as drought.

## Conclusion

Our results suggested the significant responses of species diversity and plant functional traits were found when water deficiency was alleviated along a precipitation gradient. The structural equation models demonstrated that precipitation change in amount and year has a direct effect on richness, evenness, and CWM for height, LA, SLA, DLMC, LNC and LCC, and AGB; there into, CWM for height and LDMC had a direct positive effect on AGB; LA had a direct negative effect on AGB. For dominant species, *A. polyrhizum* showed an increase in height under the precipitation treatments that promoted AGB, whereas the AGB of *P. harmala* and *S. glareosa* was boosted through alterations in height and LA. In summary, changes in precipitation amount affected plant AGB through leaf functional traits (height, LA, LDMC) rather than species diversity. We supposed that plant height, LA, LDMC are likely candidate traits, given they are mechanistically linked to precipitation changes and affected aboveground biomass in desert-steppe. And the adaptation and resource utilization strategies in response to precipitation changes are species-specific.

## Data Availability Statement

The original contributions presented in the study are included in the article/Supplementary Material, further inquiries can be directed to the corresponding author.

## Author Contributions

HC and YG conceived the idea and wrote most of the manuscript. XZ contributed to part of writing and overall improvement of the manuscript. All authors read and approved the manuscript.

## Conflict of Interest

The authors declare that the research was conducted in the absence of any commercial or financial relationships that could be construed as a potential conflict of interest.

## Publisher's Note

All claims expressed in this article are solely those of the authors and do not necessarily represent those of their affiliated organizations, or those of the publisher, the editors and the reviewers. Any product that may be evaluated in this article, or claim that may be made by its manufacturer, is not guaranteed or endorsed by the publisher.
