# Supplementary material for: Precipitation Variability Affects Aboveground Biomass Directly and Indirectly via Plant Functional Traits in the Desert Steppe of Inner Mongolia, Northern China
Source: Front Plant Sci. 2021 Aug 11;12:674527. doi: 10.3389/fpls.2021.674527 (PMC8385370; doi:10.3389/fpls.2021.674527)
Supplement: Supplementary file 1 [file Table_1.doc]

| **Table S1 Model of community-means traits selection table based on WAIC value.** The selected models for each community-means trait are highlighted in bold. | | | | | | | | |
| --- | --- | --- | --- | --- | --- | --- | --- | --- |
| CWM | Model ID | Model | WAIC | pWAIC | dWAIC | weight | SE | dSE |
| Height | **2** | **Precipitation + year** | **-199.7** | **3.9** | **0** | **0.77** | **12.28** | **NA** |
|  | 3 | Precipitation + year + Precipitation * year | -197.3 | 5 | 2.4 | 0.23 | 12.38 | 0.29 |
|  | 1 | Precipitation | -183.2 | 3.2 | 16.5 | 0 | 14.18 | 6.94 |
| Thickness | **1** | **Precipitation** | **-170.3** | **2.9** | **0** | **0.46** | **13.94** | **NA** |
|  | 2 | Precipitation + year | -170 | 3.8 | 0.3 | 0.4 | 13.21 | 2.56 |
|  | 3 | Precipitation + year + Precipitation * year | -167.8 | 4.7 | 2.4 | 0.14 | 13.16 | 2.59 |
| LA | **2** | **Precipitation + year** | **-126.7** | **4** | **0** | **0.78** | **12.29** | **NA** |
|  | 3 | Precipitation + year + Precipitation*year | -124.2 | 5.3 | 2.5 | 0.22 | 12.45 | 0.71 |
|  | 1 | Precipitation | -101.3 | 2.6 | 25.4 | 0 | 10.11 | 9.98 |
| SLA | **2** | **Precipitation + year** | **-216.6** | **4.7** | **0** | **0.76** | **16.98** | **NA** |
|  | 3 | Precipitation + year + Precipitation * year | -214.3 | 5.9 | 2.3 | 0.24 | 17.05 | 1.33 |
|  | 1 | Precipitation | -53.5 | 2.2 | 163.1 | 0 | 6.36 | 16.88 |
| LDMC | **2** | **Precipitation + year** | **-99.7** | **4.3** | **0** | **0.73** | **16.22** | **NA** |
|  | 3 | Precipitation + year + Precipitation * year | -97.7 | 5.3 | 2 | 0.27 | 16.38 | 0.93 |
|  | 1 | Precipitation | -64.4 | 2.8 | 35.3 | 0 | 12.22 | 11.74 |
| LN | **2** | **Precipitation + year** | **-97** | **4.5** | **0** | **0.68** | **16.54** | **NA** |
|  | 3 | Precipitation + year + Precipitation * year | -95.5 | 5.3 | 1.5 | 0.32 | 16.35 | 1.37 |
|  | 1 | Precipitation | -36.4 | 2.6 | 60.6 | 0 | 10.97 | 13.88 |
| LC | **2** | **Precipitation + year** | **-171.9** | **5.2** | **0** | **0.7** | **21.76** | **NA** |
|  | 3 | Precipitation + year + Precipitation * year | -170 | 6 | 1.9 | 0.27 | 21.89 | 0.65 |
|  | 1 | Precipitation | -165.4 | 3.4 | 6.5 | 0.03 | 17.26 | 7.54 |

**Table S2 Bayesian linear regression models results for traits that presented important (significant) changes in their community weighted mean values (CWM; Fig. 5 and Table S1).** We constructed three different models to investigate the drivers of changes in CWM: 1) change in CWM as a function of precipitation change, 2) as a function of the precipitation and year change, 3) and of the precipitation, year change and their interaction. The best model, shown below, was selected based on the WAIC. All Bayesian linear regression models were carried out with the CWM as response variable.

| CWM | Model ID | Model term | Mean | Std Dev | Lower 0.95 | Upper 0.95 |
| --- | --- | --- | --- | --- | --- | --- |
| Height | 2 | Intercept | 0.7 | 0.03 | 0.65 | 0.75 |
|  |  | Precipitation | 0.13 | 0.02 | 0.09 | 0.17 |
|  |  | Year | -0.07 | 0.02 | -0.1 | -0.04 |
|  |  | sigma | 0.07 | 0.01 | 0.06 | 0.08 |
| Thickness | 1 | Intercept | 0.38 | 0.01 | 0.36 | 0.39 |
|  |  | Precipitation | -0.06 | 0.02 | -0.1 | -0.01 |
|  |  | sigma | 0.09 | 0.01 | 0.07 | 0.1 |
| LA | 2 | Intercept | 0.3 | 0.04 | 0.23 | 0.38 |
|  |  | Precipitation | 0.17 | 0.03 | 0.11 | 0.23 |
|  |  | Year | 0.14 | 0.02 | 0.09 | 0.18 |
|  |  | sigma | 0.11 | 0.01 | 0.09 | 0.13 |
| SLA | 2 | Intercept | 0.2 | 0.02 | 0.15 | 0.24 |
|  |  | Precipitation | -0.03 | 0.02 | -0.07 | 0 |
|  |  | Year | 0.32 | 0.01 | 0.29 | 0.35 |
|  |  | sigma | 0.07 | 0.01 | 0.06 | 0.08 |
| LDMC | 2 | Intercept | 0.91 | 0.04 | 0.82 | 1 |
|  |  | Precipitation | 0.12 | 0.04 | 0.05 | 0.19 |
|  |  | Year | -0.19 | 0.03 | -0.25 | -0.14 |
|  |  | sigma | 0.13 | 0.01 | 0.11 | 0.15 |
| LN | 2 | Intercept | 0.23 | 0.05 | 0.15 | 0.33 |
|  |  | Precipitation | -0.18 | 0.04 | -0.25 | -0.11 |
|  |  | Year | 0.27 | 0.03 | 0.22 | 0.33 |
|  |  | sigma | 0.13 | 0.01 | 0.11 | 0.16 |
| LC | 2 | Intercept | 0.84 | 0.03 | 0.78 | 0.89 |
|  |  | Precipitation | 0.09 | 0.02 | 0.04 | 0.13 |
|  |  | Year | -0.06 | 0.02 | -0.09 | -0.02 |
|  |  | sigma | 0.08 | 0.01 | 0.07 | 0.1 |

**Table S3 Model of traits selection table based on WAIC value.** The selected models for each trait are highlighted in bold.

| Trait | Model ID | Model | WAIC | pWAIC | dWAIC | weight | SE | dSE |
| --- | --- | --- | --- | --- | --- | --- | --- | --- |
| Coverage | **5** | **[spp]* precipitation + [spp] * year** | **-166** | **12.2** | **0** | **0.61** | **33.7** | **NA** |
|  | 6 | [spp]* precipitation + [spp] * year + [spp]*precipitation*year | -165 | 13.8 | 0.9 | 0.39 | 33.5 | 2.84 |
|  | 3 | Precipitation + year + Precipitation*year | -126 | 5.6 | 40 | 0 | 26.1 | 16 |
|  | 2 | Precipitation + year | -123 | 5 | 43 | 0 | 27.7 | 15.4 |
|  | 4 | [spp]* precipitation | -108 | 9.4 | 57.9 | 0 | 32.6 | 12.2 |
|  | 1 | Precipitation | -81 | 4.2 | 85.3 | 0 | 28 | 19.3 |
| Height | **5** | **[spp]* precipitation + [spp] * year** | **-315** | **11.6** | **0** | **0.7** | **41.6** | **NA** |
|  | 6 | [spp]* precipitation + [spp] * year + [spp]*precipitation*year | -313 | 13.6 | 1.8 | 0.29 | 40.8 | 2.79 |
|  | 2 | Precipitation + year | -304 | 6.1 | 10.9 | 0 | 37 | 8.66 |
|  | 3 | Precipitation + year + Precipitation*year | -303 | 6.9 | 12.6 | 0 | 36.7 | 8.92 |
|  | 4 | [spp]* precipitation | -293 | 9.4 | 21.8 | 0 | 44.3 | 9.45 |
|  | 1 | Precipitation | -289 | 5.2 | 26.2 | 0 | 37.2 | 11.4 |
| Thickness | **5** | **[spp]* precipitation + [spp] * year** | **-239** | **10** | **0** | **0.54** | **27.2** | **NA** |
|  | 6 | [spp]* precipitation + [spp] * year + [spp]*precipitation*year | -238 | 11.8 | 0.3 | 0.46 | 27.6 | 3.2 |
|  | 4 | [spp]* precipitation | -208 | 6.8 | 30.5 | 0 | 26 | 10.1 |
|  | 1 | Precipitation | -145 | 3.5 | 93.6 | 0 | 25.5 | 17.7 |
|  | 2 | Precipitation + year | -144 | 4.5 | 94.8 | 0 | 25.8 | 17.7 |
|  | 3 | Precipitation + year + Precipitation*year | -143 | 5.2 | 96.2 | 0 | 25.9 | 17.7 |
| LA | **5** | **[spp]* precipitation + [spp] * year** | **-437** | **13.7** | **0** | **0.85** | **42.5** | **NA** |
|  | 6 | [spp]* precipitation + [spp] * year + [spp]*precipitation*year | -433 | 17.7 | 3.4 | 0.15 | 41.9 | 2.36 |
|  | 4 | [spp]* precipitation | -225 | 7 | 211.9 | 0 | 25.7 | 28.7 |
|  | 2 | Precipitation + year | -22.1 | 3.3 | 414.7 | 0 | 9.97 | 42.8 |
|  | 3 | Precipitation + year + Precipitation*year | -20.2 | 4.2 | 416.6 | 0 | 9.99 | 42.8 |
|  | 1 | Precipitation | 10.3 | 2.4 | 447.1 | 0 | 12.8 | 42.8 |
| SLA | **6** | **[spp]* precipitation + [spp] * year + [spp]*precipitation*year** | **-402** | **18.7** | **0** | **0.98** | **44.3** | **NA** |
|  | 5 | [spp]* precipitation + [spp] * year | -393 | 13.8 | 8.2 | 0.02 | 43.1 | 6.71 |
|  | 2 | Precipitation + year | -326 | 5.8 | 76 | 0 | 31.6 | 21.4 |
|  | 3 | Precipitation + year + Precipitation*year | -323 | 7.2 | 78.4 | 0 | 31.9 | 21.4 |
|  | 4 | [spp]* precipitation | -125 | 6.2 | 276.2 | 0 | 18.5 | 37.4 |
|  | 1 | Precipitation | -97 | 2.6 | 304.5 | 0 | 14.7 | 40.5 |
| LDMC | **6** | **[spp]* precipitation + [spp] * year + [spp]*precipitation*year** | **-295** | **18** | **0** | **0.6** | **49.2** | **NA** |
|  | 5 | [spp]* precipitation + [spp] * year | -294 | 15.3 | 0.8 | 0.4 | 50.1 | 3.65 |
|  | 4 | [spp]* precipitation | -270 | 11.1 | 25.2 | 0 | 42.4 | 10.7 |
|  | 2 | Precipitation + year | -36.6 | 3.6 | 258.2 | 0 | 14 | 46.2 |
|  | 3 | Precipitation + year + Precipitation*year | -34.7 | 4.5 | 260.1 | 0 | 13.9 | 46.2 |
|  | 1 | precipitation | -14.1 | 2.5 | 280.7 | 0 | 14 | 47.1 |
| LN | **6** | **[spp]* precipitation + [spp] * year + [spp]*precipitation*year** | **-389** | **13.2** | **0** | **0.61** | **26.9** | **NA** |
|  | 5 | [spp]* precipitation + [spp] * year | -388 | 10.6 | 0.9 | 0.39 | 26.3 | 6.41 |
|  | 4 | [spp]* precipitation | -207 | 6.3 | 182.1 | 0 | 22.4 | 23.7 |
|  | 2 | Precipitation + year | -92.9 | 4.3 | 295.8 | 0 | 19.3 | 23.8 |
|  | 3 | Precipitation + year + Precipitation*year | -90.6 | 5.6 | 298.1 | 0 | 19.4 | 23.8 |
|  | 1 | Precipitation | -29.5 | 3.2 | 359.2 | 0 | 20.4 | 25.1 |
| LC | **4** | **[spp]* precipitation** | **-366** | **18.2** | **0** | **0.88** | **82.1** | **NA** |
|  | 5 | [spp]* precipitation + [spp] * year | -361 | 20.8 | 4.7 | 0.08 | 82.3 | 1.78 |
|  | 6 | [spp]* precipitation + [spp] * year + [spp]*precipitation*year | -359 | 21.7 | 6.8 | 0.03 | 81.8 | 1.95 |
|  | 2 | Precipitation + year | -209 | 4.6 | 156.8 | 0 | 25.2 | 63.8 |
|  | 1 | Precipitation | -208 | 3.3 | 157.6 | 0 | 23.2 | 65.7 |
|  | 3 | Precipitation + year + Precipitation*year | -207 | 5.5 | 158.9 | 0 | 25.1 | 63.8 |

**Table S4 Bayesian linear regression models results for traits that presented important (significant) changes in dominant species. We constructed six different models to investigate the drivers of changes in trait:** 1)change in CWM as a function of precipitation change, 2) as a function of the precipitation and year change, 3) and of the precipitation, year change and their interaction, 4) change in CWM as a function of precipitation change and species as random effects, 5) as a function of the precipitation and year change, and species as random effects, 6) of the precipitation, year change and their interaction and species as random effects. The best model, shown below, was selected based on the WAIC. All Bayesian linear regression models were carried out with the trait value as response variable, *St* is *Stipa glareosa,* *Pe* is *Peganum harmala,* *Al*1 is *Allium polyrhizum.*

| Trait | Model ID | Model term | Mean | Std Dev | lower 0.95 | upper 0.95 |
| --- | --- | --- | --- | --- | --- | --- |
| Coverage | 5 | Intercept | 0.78 | 0.5 | -0.17 | 1.73 |
|  |  | Intercept [*St*] | 1.03 | 0.06 | 0.92 | 1.15 |
|  |  | Intercept [*Pe*] | 0.59 | 0.06 | 0.46 | 0.7 |
|  |  | Intercept [*Al*1] | 0.74 | 0.09 | 0.57 | 0.92 |
|  |  | Year | -0.16 | 0.38 | -0.82 | 0.56 |
|  |  | Year [*St*] | -0.28 | 0.04 | -0.35 | -0.21 |
|  |  | Year [*Pe*] | -0.03 | 0.04 | -0.1 | 0.04 |
|  |  | Year [*Al*1] | -0.17 | 0.05 | -0.27 | -0.07 |
|  |  | Precipitation | 0.31 | 0.42 | -0.36 | 1 |
|  |  | Precipitation [*St*] | 0.42 | 0.05 | 0.31 | 0.52 |
|  |  | Precipitation [*Pe*] | 0.3 | 0.04 | 0.21 | 0.38 |
|  |  | Precipitation [*Al*1] | 0.2 | 0.06 | 0.08 | 0.33 |
|  |  | Sigma | 0.15 | 0.01 | 0.13 | 0.16 |
| Height | 5 | Intercept | 0.66 | 0.38 | 0.01 | 1.3 |
|  |  | Intercept [*St*] | 0.79 | 0.04 | 0.71 | 0.87 |
|  |  | Intercept [*Pe*] | 0.61 | 0.04 | 0.54 | 0.69 |
|  |  | Intercept [*Al*1] | 0.57 | 0.06 | 0.45 | 0.69 |
|  |  | Year | -0.07 | 0.31 | -0.43 | 0.33 |
|  |  | Year [*St*] | -0.12 | 0.03 | -0.17 | -0.07 |
|  |  | Year [*Pe*] | -0.03 | 0.02 | -0.08 | 0.01 |
|  |  | Year [*Al*1] | -0.01 | 0.03 | -0.07 | 0.06 |
|  |  | Precipitation | 0.11 | 0.17 | -0.08 | 0.3 |
|  |  | Precipitation [*St*] | 0.11 | 0.03 | 0.06 | 0.17 |
|  |  | Precipitation [*Pe*] | 0.1 | 0.02 | 0.05 | 0.14 |
|  |  | Precipitation [*Al*1] | 0.11 | 0.03 | 0.04 | 0.17 |
|  |  | Sigma | 0.1 | 0.01 | 0.09 | 0.11 |
| Thickness | 5 | Intercept | 0.47 | 0.37 | -0.04 | 0.95 |
|  |  | Intercept [*St*] | 0.54 | 0.05 | 0.44 | 0.63 |
|  |  | Intercept [*Pe*] | 0.4 | 0.05 | 0.31 | 0.5 |
|  |  | Intercept [*Al*1] | 0.46 | 0.07 | 0.32 | 0.59 |
|  |  | Year | -0.05 | 0.35 | -0.68 | 0.57 |
|  |  | Year [*St*] | -0.17 | 0.03 | -0.24 | -0.11 |
|  |  | Year [*Pe*] | -0.02 | 0.03 | -0.07 | 0.04 |
|  |  | Year [*Al*1] | 0.04 | 0.04 | -0.04 | 0.11 |
|  |  | Precipitation | -0.02 | 0.27 | -0.31 | 0.3 |
|  |  | Precipitation [*St*] | 0.01 | 0.04 | -0.06 | 0.09 |
|  |  | Precipitation [*Pe*] | -0.03 | 0.03 | -0.09 | 0.03 |
|  |  | Precipitation [*Al*1] | -0.02 | 0.04 | -0.1 | 0.06 |
|  |  | Sigma | 0.12 | 0.01 | 0.11 | 0.13 |
| LA | 5 | Intercept | 0.2 | 0.44 | -0.6 | 1.04 |
|  |  | Intercept [*St*] | 0.37 | 0.03 | 0.32 | 0.42 |
|  |  | Intercept [*Pe*] | 0.03 | 0.03 | -0.02 | 0.09 |
|  |  | Intercept [*Al*1] | 0.21 | 0.04 | 0.13 | 0.29 |
|  |  | Year | 0.23 | 0.36 | -0.29 | 0.76 |
|  |  | Year [*St*] | 0.22 | 0.02 | 0.18 | 0.25 |
|  |  | Year [*Pe*] | 0.16 | 0.02 | 0.12 | 0.19 |
|  |  | Year [*Al*1] | 0.32 | 0.02 | 0.27 | 0.36 |
|  |  | Precipitation | 0.02 | 0.11 | -0.13 | 0.19 |
|  |  | Precipitation [*St*] | 0.02 | 0.02 | -0.02 | 0.06 |
|  |  | Precipitation [*Pe*] | 0.02 | 0.02 | -0.02 | 0.06 |
|  |  | Precipitation [*Al*1] | 0.04 | 0.03 | -0.01 | 0.09 |
|  |  | Sigma | 0.07 | 0 | 0.06 | 0.08 |
| SLA | 6 | Intercept | 0.21 | 0.4 | -0.56 | 0.99 |
|  |  | Intercept [*St*] | 0.06 | 0.03 | 0 | 0.12 |
|  |  | Intercept [*Pe*] | 0.4 | 0.03 | 0.33 | 0.46 |
|  |  | Intercept [*Al*1] | 0.19 | 0.05 | 0.1 | 0.28 |
|  |  | Year * Precipitation | -0.08 | 0.35 | -0.69 | 0.51 |
|  |  | Year * Precipitation [St] | -0.18 | 0.06 | -0.28 | -0.06 |
|  |  | Year * Precipitation [*Pe*] | 0.03 | 0.05 | -0.07 | 0.12 |
|  |  | Year * Precipitation [*Al*1] | -0.11 | 0.06 | -0.24 | 0.02 |
|  |  | Year | 0.31 | 0.31 | -0.22 | 0.88 |
|  |  | Year [*St*] | 0.39 | 0.02 | 0.35 | 0.43 |
|  |  | Year [*Pe*] | 0.21 | 0.02 | 0.17 | 0.24 |
|  |  | Year [*Al*1] | 0.36 | 0.03 | 0.31 | 0.41 |
|  |  | Precipitation | 0.1 | 0.42 | -0.72 | 0.89 |
|  |  | Precipitation [*St*] | 0.21 | 0.08 | 0.05 | 0.37 |
|  |  | Precipitation [*Pe*] | -0.07 | 0.08 | -0.22 | 0.09 |
|  |  | Precipitation [*Al*1] | 0.18 | 0.12 | -0.04 | 0.42 |
|  |  | Sigma | 0.07 | 0 | 0.07 | 0.08 |
| LDMC | 6 | Intercept | 0.68 | 0.68 | -0.69 | 1.95 |
|  |  | Intercept [*St*] | 1.03 | 0.04 | 0.95 | 1.12 |
|  |  | Intercept [*Pe*] | 0.64 | 0.04 | 0.56 | 0.72 |
|  |  | Intercept [*Al*1] | 0.38 | 0.06 | 0.25 | 0.5 |
|  |  | Precipitation * year | 0.05 | 0.36 | -0.51 | 0.59 |
|  |  | Year * Precipitation [*St*] | 0.06 | 0.06 | -0.07 | 0.18 |
|  |  | Year * Precipitation [*Pe*] | -0.03 | 0.06 | -0.14 | 0.09 |
|  |  | Year * Precipitation [*Al*1] | 0.13 | 0.09 | -0.03 | 0.32 |
|  |  | Year | -0.08 | 0.24 | -0.46 | 0.33 |
|  |  | Year [*St*] | -0.13 | 0.03 | -0.19 | -0.08 |
|  |  | Year [*Pe*] | -0.06 | 0.02 | -0.11 | -0.02 |
|  |  | Year [*Al*1] | -0.03 | 0.04 | -0.1 | 0.03 |
|  |  | Precipitation | -0.02 | 0.32 | -0.66 | 0.64 |
|  |  | Precipitation [*St*] | -0.01 | 0.09 | -0.2 | 0.17 |
|  |  | Precipitation [*Pe*] | 0.05 | 0.1 | -0.13 | 0.24 |
|  |  | Precipitation [*Al*1] | -0.12 | 0.17 | -0.46 | 0.18 |
|  |  | Sigma | 0.1 | 0.01 | 0.09 | 0.11 |
| LN | 6 | Intercept | 0.44 | 0.77 | -0.99 | 1.91 |
|  |  | Intercept [*St*] | -0.05 | 0.03 | -0.12 | 0.01 |
|  |  | Intercept [*Pe*] | 0.56 | 0.03 | 0.5 | 0.62 |
|  |  | Intercept [*Al*1] | 0.86 | 0.05 | 0.76 | 0.96 |
|  |  | Year*Precipitation | -0.07 | 0.19 | -0.37 | 0.25 |
|  |  | Year * Precipitation [*St*] | -0.1 | 0.05 | -0.2 | -0.01 |
|  |  | Year * Precipitation [*Pe*] | -0.05 | 0.04 | -0.13 | 0.03 |
|  |  | Year * Precipitation [*Al*1] | -0.06 | 0.06 | -0.16 | 0.06 |
|  |  | Year | 0.17 | 0.45 | -0.58 | 0.95 |
|  |  | Year [*St*] | 0.34 | 0.02 | 0.3 | 0.38 |
|  |  | Year [*Pe*] | 0.13 | 0.02 | 0.1 | 0.17 |
|  |  | Year [*Al*1] | 0 | 0.03 | -0.05 | 0.06 |
|  |  | Precipitation | 0.02 | 0.27 | -0.45 | 0.49 |
|  |  | Precipitation [*St*] | 0.05 | 0.07 | -0.09 | 0.19 |
|  |  | Precipitation [*Pe*] | 0.04 | 0.07 | -0.09 | 0.17 |
|  |  | Precipitation [*Al*1] | -0.04 | 0.1 | -0.26 | 0.15 |
|  |  | Sigma | 0.08 | 0 | 0.07 | 0.09 |
| LC | 4 | Intercept | 0.74 | 0.37 | 0.07 | 1.4 |
|  |  | Intercept [*St*] | 0.89 | 0.01 | 0.87 | 0.91 |
|  |  | Intercept [*Pe*] | 0.66 | 0.01 | 0.64 | 0.68 |
|  |  | Intercept [*Al*1] | 0.69 | 0.01 | 0.66 | 0.71 |
|  |  | Precipitation | 0.02 | 0.19 | -0.24 | 0.27 |
|  |  | Precipitation [*St*] | 0.05 | 0.03 | 0 | 0.11 |
|  |  | Precipitation [*Pe*] | 0 | 0.02 | -0.05 | 0.04 |
|  |  | Precipitation [*Al*1] | 0.01 | 0.03 | -0.05 | 0.07 |
|  |  | Sigma | 0.08 | 0 | 0.07 | 0.09 |

**Table S5 All the species in this study**

| Species | Latin Name | Family |  | Lifestyle | Vegetation type |
| --- | --- | --- | --- | --- | --- |
| *A. splendens* | *Achnatherum splendens*(Trin.) Nevski | Gramineae |  | Perennial | Herb |
| *A. cristatum* | *Agropyron cristatum* (L.) Gaertn. | Gramineae |  | Perennial | Herb |
| *A. sparsifolia* | *Alhagi sparsifolia* Shap. | Leguminosae | | Perennial | Subshrub |
| *A. mongolicum* | *Allium mongolicum*Regel | Liliaceae |  | Perennial | Herb |
| *A. polyrhizum* | *Allium polyrhizum* Turcz. ex Regel | Liliaceae |  | Perennial | Herb |
| *A. frigida* | *Artemisia frigida* Willd. | Compositae | | Perennial | Herb |
| *A. cochinchinensis* | *Asparagus cochinchinensis* (Lour.) Merr. | Liliaceae |  | Perennial | Herb |
| *A. gobicus* | *Asparagus gobicus* Ivan. ex Grubov | Liliaceae |  | Perennial | Subshrub |
| *A. scaberrimus* | *Astragalus scaberrimus* Bunge | Leguminosae | | Perennial | Herb |
| *B. dasyphylla* | *Bassia dasyphylla* (Fisch. et C. A. Mey.) Kuntze | Chenopodiaceae | | Annual | Herb |
| *C. latens* | *Krascheninnikovia latens* (J. F. Gmel.) Reveal et Holmgren | Chenopodiaceae | | Perennial | Shrub |
| *C. sabulosa* | *Chamaerhodos sabulosa* Bge. | Rosaceae |  | Perennial | Herb |
| *C. squarrosa* | *Cleistogenes squarrosa* (Trin.) Keng | Gramineae |  | Perennial | Herb |
| *C. ammannii* | *Convolvulus ammannii* Desr. | Convolvulaceae | | Perennial | Herb |
| *C. drummondii* | *Coreopsis drummondii*Torr. et Gray | Asteraceae |  | Annual | Herb |
| *C. mongolicum* | *Corispermum mongolicum* Iljin | Amaranthaceae | | Annual | Herb |
| *E. pilosa* | *Eragrostis pilosa* (L.) Beauv. | Gramineae |  | Annual | Herb |
| *F. bungeana* | *Ferula bungeana* Kitagawa | Apiaceae |  | Perennial | Herb |
| *G. elegans* | *Gypsophila elegans* M. Bieb. | Caryophyllaceae | | Annual | Herb |
| *H. hispidus* | *Heteropappus hispidus* (Thunb.) Less. | Asteraceae |  | Annual | Herb |
| *K. prostrata* | *Kochia prostrata* (L.) Schrad. | Chenopodiaceae | | Annual/Perennial | Herb |
| *L. lanatonodus* | *Lagochilus lanatonodus* C. Y. Wu et Hsuan | Lamiaceae |  | Perennial | Herb |
| *N. pectinata* | *Neopallasia pectinata* (Pallas) Poljakov | Asteraceae |  | Annual | Herb |
| *O. aciphylla* | *Oxytropis aciphylla* Ledeb. | Leguminosae | | Perennial | Subshrub |
| *O. platysema* | *Oxytropis platysema* Schrenk | Leguminosae | | Perennial | Herb |
| *P. harmala* | *Peganum harmala* L. | Nitrariaceae | | Perennial | Herb |
| *P. minuta* | *Plantago minuta* Pall. | Plantaginaceae | | Annual/Perennial | Herb |
| *P. canescens* | *Ptilotricum canescens* (DC.) C. A. Mey. | Brassicaceae | | Perennial | Subshrub |
| *R. songarica* | *Reaumuria songarica* (Pall.) Maxim. | Tamaricaceae | | Perennial | Shrub |
| *S. collina* | *Salsola collina* Pall. | Amaranthaceae | | Annual | Herb |
| *S. divaricata* | *Saposhnikovia divaricata* (Turcz.) Schischk. | Apiaceae |  | Perennial | Herb |
| *S. albicaulis* | *Scorzonera albicaulis* Bunge | Asteraceae |  | Perennial | Herb |
| *S. divaricata* | *Scorzonera divaricata* Turcz. | Asteraceae |  | Perennial | Herb |
| *S. pseudodivaricata* | *Scorzonera pseudodivaricata* Lipschitz | Asteraceae |  | Perennial | Herb |
| *S. viridis* | *Setaria viridis* (L.) Beauv. | Gramineae |  | Annual | Herb |
| *S. glareosa* | *Stipa glareosa* P. Smirn. | Gramineae |  | Perennial | Herb |

**Table S6 Pearson correlation analysis in 2017**

|  | precipitation | Nheight | thickness | Nla | Nsla | Nldmc | Nn | c | Nbimass | diversity | evenness |
| --- | --- | --- | --- | --- | --- | --- | --- | --- | --- | --- | --- |
| Nheight | .541^**^ |  |  |  |  |  |  |  |  |  |  |
| thickness | -0.184 | -.464^**^ |  |  |  |  |  |  |  |  |  |
| Nla | .495^**^ | .454^**^ | -0.184 |  |  |  |  |  |  |  |  |
| Nsla | -0.195 | -.503^**^ | .316^*^ | -0.121 |  |  |  |  |  |  |  |
| Nldmc | .347^*^ | .656^**^ | -.478^**^ | 0.280 | -.828^**^ |  |  |  |  |  |  |
| Nn | -.440^**^ | -.756^**^ | .467^**^ | -.474^**^ | .679^**^ | -.865^**^ |  |  |  |  |  |
| c | .447^**^ | .547^**^ | -.325^*^ | .453^**^ | -.468^**^ | .566^**^ | -.587^**^ |  |  |  |  |
| Nbimass | .632^**^ | .728^**^ | -.381^*^ | 0.189 | -.491^**^ | .592^**^ | -.564^**^ | .525^**^ |  |  |  |
| diversity | 0.069 | -0.026 | 0.001 | -0.211 | .320^*^ | -0.145 | 0.034 | -0.203 | -0.005 |  |  |
| evenness | -.468^**^ | -.429^**^ | .306^*^ | -.469^**^ | .408^**^ | -.575^**^ | .525^**^ | -.413^**^ | -.406^**^ | .560^**^ |  |
| Nrichnes | .592^**^ | .388^*^ | -0.270 | 0.283 | -0.053 | .394^**^ | -.470^**^ | 0.179 | .401^**^ | .496^**^ | -.426^**^ |

** means *p*< 0.01

* means *p*< 0.05

**Table S7 Pearson correlation analysis in 2018**

|  | precipitation | Nheight | thickness | Nla | Nsla | Nldmc | Nn | c | Nbimass | diversity | evenness |
| --- | --- | --- | --- | --- | --- | --- | --- | --- | --- | --- | --- |
| Nheight | .691^**^ |  |  |  |  |  |  |  |  |  |  |
| thickness | -0.211 | -0.156 |  |  |  |  |  |  |  |  |  |
| Nla | .431^**^ | .391^*^ | 0.153 |  |  |  |  |  |  |  |  |
| Nsla | -0.253 | -.458^**^ | 0.159 | 0.153 |  |  |  |  |  |  |  |
| Nldmc | .478^**^ | .509^**^ | -.550^**^ | 0.170 | -.647^**^ |  |  |  |  |  |  |
| Nn | -.641^**^ | -.545^**^ | .407^**^ | -0.292 | .598^**^ | -.811^**^ |  |  |  |  |  |
| c | .656^**^ | .626^**^ | -0.292 | .588^**^ | -.419^**^ | .692^**^ | -.651^**^ |  |  |  |  |
| Nbimass | .662^**^ | .475^**^ | -0.223 | 0.179 | -0.175 | .441^**^ | -.510^**^ | .314^*^ |  |  |  |
| diversity | .321^*^ | 0.152 | 0.040 | .412^**^ | -0.217 | .378^*^ | -.512^**^ | .494^**^ | .356^*^ |  |  |
| evenness | -.513^**^ | -.373^*^ | 0.255 | -0.037 | 0.052 | -0.214 | 0.120 | -0.169 | -0.274 | .480^**^ |  |
| Nrichnes | .794^**^ | .523^**^ | -0.196 | .453^**^ | -.361^*^ | .619^**^ | -.680^**^ | .688^**^ | .603^**^ | .593^**^ | -.392^*^ |

** means *p*< 0.01

* means *p*< 0.05

**Table S8 Mean and SE of plant functional traits under precipitation treatments in 2017 and 2018**

| Functional Trait | Treatment | 2017 | |  | 2018 | |
| --- | --- | --- | --- | --- | --- | --- |
|  |  | Mean | SE |  | Mean | SE |
| Height | -0.6 | -0.29 | 0.47 |  | -1.09 | 0.27 |
|  | -0.4 | -0.68 | 0.26 |  | -1.20 | 0.27 |
|  | -0.2 | 0.19 | 0.54 |  | -0.49 | 0.29 |
|  | 0 | 0.46 | 0.28 |  | -0.48 | 0.12 |
|  | 0.2 | 0.90 | 0.33 |  | 0.28 | 0.26 |
|  | 0.4 | 0.53 | 0.20 |  | 0.31 | 0.26 |
|  | 0.6 | 1.20 | 0.30 |  | 0.35 | 0.27 |
| thickness | -0.6 | 0.53 | 0.03 |  | 0.48 | 0.03 |
|  | -0.4 | 0.54 | 0.03 |  | 0.58 | 0.03 |
|  | -0.2 | 0.48 | 0.04 |  | 0.46 | 0.06 |
|  | 0 | 0.46 | 0.02 |  | 0.46 | 0.02 |
|  | 0.2 | 0.56 | 0.05 |  | 0.48 | 0.06 |
|  | 0.4 | 0.46 | 0.04 |  | 0.48 | 0.01 |
|  | 0.6 | 0.48 | 0.05 |  | 0.45 | 0.04 |
| LA | -0.6 | -1.18 | 0.35 |  | -0.45 | 0.49 |
|  | -0.4 | -1.17 | 0.32 |  | 0.10 | 0.37 |
|  | -0.2 | -0.26 | 0.27 |  | 0.69 | 0.27 |
|  | 0 | -0.20 | 0.10 |  | 0.35 | 0.34 |
|  | 0.2 | -0.36 | 0.13 |  | 1.33 | 0.33 |
|  | 0.4 | -0.35 | 0.12 |  | 0.78 | 0.21 |
|  | 0.6 | -0.09 | 0.28 |  | 0.81 | 0.40 |
| SLA | -0.6 | -0.88 | 0.36 |  | 0.31 | 0.07 |
|  | -0.4 | -0.39 | 0.29 |  | 1.21 | 0.33 |
|  | -0.2 | -0.41 | 0.29 |  | 1.28 | 0.23 |
|  | 0 | -0.88 | 0.16 |  | 1.00 | 0.21 |
|  | 0.2 | -1.00 | 0.19 |  | 0.91 | 0.22 |
|  | 0.4 | -0.68 | 0.08 |  | 0.29 | 0.14 |
|  | 0.6 | -1.07 | 0.32 |  | 0.33 | 0.09 |
| LDMC | -0.6 | 0.43 | 0.52 |  | -0.49 | 0.17 |
|  | -0.4 | -0.34 | 0.47 |  | -1.28 | 0.33 |
|  | -0.2 | -0.13 | 0.48 |  | -0.65 | 0.26 |
|  | 0 | 0.82 | 0.26 |  | -0.54 | 0.16 |
|  | 0.2 | 0.64 | 0.59 |  | -0.29 | 0.21 |
|  | 0.4 | 0.70 | 0.12 |  | 0.06 | 0.11 |
|  | 0.6 | 1.18 | 0.22 |  | -0.12 | 0.18 |
| LNC | -0.6 | -0.30 | 0.27 |  | 0.64 | 0.19 |
|  | -0.4 | 0.23 | 0.52 |  | 1.46 | 0.23 |
|  | -0.2 | -0.08 | 0.44 |  | 0.96 | 0.29 |
|  | 0 | -0.77 | 0.22 |  | 0.59 | 0.10 |
|  | 0.2 | -0.79 | 0.45 |  | 0.37 | 0.15 |
|  | 0.4 | -1.04 | 0.20 |  | -0.02 | 0.08 |
|  | 0.6 | -1.16 | 0.33 |  | -0.10 | 0.12 |
| LCC | -0.6 | 43.19 | 0.40 |  | 42.03 | 0.18 |
|  | -0.4 | 42.40 | 0.94 |  | 41.83 | 0.28 |
|  | -0.2 | 42.52 | 1.21 |  | 42.52 | 0.32 |
|  | 0 | 44.78 | 0.48 |  | 42.52 | 0.45 |
|  | 0.2 | 44.72 | 0.53 |  | 43.31 | 0.30 |
|  | 0.4 | 44.22 | 0.50 |  | 43.63 | 0.21 |
|  | 0.6 | 45.16 | 0.28 |  | 43.54 | 0.37 |

**Table S9 Information of structural equation models (SEM) of precipitation and year effect on aboveground biomass of community**

| Probability level = .628 | | | | | | | | | | |
| --- | --- | --- | --- | --- | --- | --- | --- | --- | --- | --- |
| Standardized Direct Effects | | | | | | | | | | |
|  | Year | Precipitation | LCC | LNC | LDMC | SLA | LA | Height | Evenness | Richness |
| LCC | 0.173 | 0.506 | 0 | 0 | 0 | 0 | 0 | 0 | 0 | 0 |
| LNC | -0.418 | -0.447 | 0 | 0 | 0 | 0 | 0 | 0 | 0 | 0 |
| LDMC | 0.362 | 0.364 | 0 | 0 | 0 | 0 | 0 | 0 | 0 | 0 |
| SLA | -0.713 | -0.144 | 0 | 0 | 0 | 0 | 0 | 0 | 0 | 0 |
| LA | -0.661 | 0.399 | 0 | 0 | 0 | 0 | 0 | 0 | 0 | 0 |
| Height | 0.131 | 0.599 | 0 | 0 | 0 | 0 | 0 | 0 | 0 | 0 |
| Evenness | -0.111 | -0.494 | 0 | 0 | 0 | 0 | 0 | 0 | 0 | 0 |
| Richness | -0.382 | 0.706 | 0 | 0 | 0 | 0 | 0 | 0 | 0 | 0 |
| Aboveground biomass | -0.005 | 0.448 | 0.029 | 0.059 | 0.37 | 0.239 | -0.259 | 0.322 | 0.088 | 0.072 |
| Standardized Indirect Effects | |  |  |  |  |  |  |  |  |  |
|  | Year | Precipitation | LCC | LNC | LDMC | SLA | LA | Height | Evenness | Richness |
| LCC | 0 | 0 | 0 | 0 | 0 | 0 | 0 | 0 | 0 | 0 |
| LNC | 0 | 0 | 0 | 0 | 0 | 0 | 0 | 0 | 0 | 0 |
| LDMC | 0 | 0 | 0 | 0 | 0 | 0 | 0 | 0 | 0 | 0 |
| SLA | 0 | 0 | 0 | 0 | 0 | 0 | 0 | 0 | 0 | 0 |
| LA | 0 | 0 | 0 | 0 | 0 | 0 | 0 | 0 | 0 | 0 |
| Height | 0 | 0 | 0 | 0 | 0 | 0 | 0 | 0 | 0 | 0 |
| Evenness | 0 | 0 | 0 | 0 | 0 | 0 | 0 | 0 | 0 | 0 |
| Richness | 0 | 0 | 0 | 0 | 0 | 0 | 0 | 0 | 0 | 0 |
| Aboveground biomass | 0.12 | 0.185 | 0 | 0 | 0 | 0 | 0 | 0 | 0 | 0 |

**Table S10 Information of structural equation models (SEM) of precipitation and year effect on aboveground biomass of *A. polyrhizum***

| Probability level = .537 | |  |  |  |  |  |  |
| --- | --- | --- | --- | --- | --- | --- | --- |
| Standardized Direct Effects | |  |  |  |  |  |  |
|  | Precipitation | Year | LDMC | SLA | LNC | Height | LA |
| LDMC | 0.211 | 0 | 0 | 0 | 0 | 0 | 0 |
| SLA | 0 | -0.718 | 0 | 0 | 0 | 0 | 0 |
| LNC | -0.816 | 0.3 | 0 | 0 | 0 | 0 | 0 |
| Height | 0.33 | 0 | 0 | 0 | 0 | 0 | 0 |
| LA | 0 | -0.762 | 0 | 0 | 0 | 0 | 0 |
| Aboveground biomass | 0.324 | -0.035 | -0.163 | 0.183 | 0.144 | 0.37 | -0.413 |
| Standardized Indirect Effects | |  |  |  |  |  |  |
|  | Precipitation | Year | LDMC | SLA | LNC | Height | LA |
| LDMC | 0 | 0 | 0 | 0 | 0 | 0 | 0 |
| SLA | 0 | 0 | 0 | 0 | 0 | 0 | 0 |
| LNC | 0 | 0 | 0 | 0 | 0 | 0 | 0 |
| Height | 0 | 0 | 0 | 0 | 0 | 0 | 0 |
| LA | 0 | 0 | 0 | 0 | 0 | 0 | 0 |
| Aboveground biomass | -0.03 | 0.226 | 0 | 0 | 0 | 0 | 0 |

**Table S11 Information of structural equation models (SEM) of precipitation and year effect on aboveground biomass of *P. harmala***

| Probability level = .341 | |  |  |  |  |  |  |
| --- | --- | --- | --- | --- | --- | --- | --- |
| Standardized Direct Effects | |  |  |  |  |  |  |
|  | Year | Precipitation | LCC | LNC | SLA | LA | Height |
| LCC | 0.275 | 0 | 0 | 0 | 0 | 0 | 0 |
| LNC | -0.622 | 0 | 0 | 0 | 0 | 0 | 0 |
| SLA | -0.695 | 0 | 0 | 0 | 0 | 0 | 0 |
| LA | -0.708 | 0 | 0 | 0 | 0 | 0 | 0 |
| Height | 0 | 0.454 | 0 | 0 | 0 | 0 | 0 |
| Aboveground biomass | 0.141 | 0.117 | -0.006 | -0.011 | -0.061 | 0.335 | 0.439 |
| **Standardized Indirect Effects** | |  |  |  |  |  |  |
|  | Year | Precipitation | LCC | LNC | SLA | LA | Height |
| LCC | 0 | 0 | 0 | 0 | 0 | 0 | 0 |
| LNC | 0 | 0 | 0 | 0 | 0 | 0 | 0 |
| SLA | 0 | 0 | 0 | 0 | 0 | 0 | 0 |
| LA | 0 | 0 | 0 | 0 | 0 | 0 | 0 |
| Height | 0 | 0 | 0 | 0 | 0 | 0 | 0 |
| Aboveground biomass | -0.19 | 0.199 | 0 | 0 | 0 | 0 | 0 |

**Table S12 Information of structural equation models (SEM) of precipitation and year effect on aboveground biomass of *S. glareosa:***

| Probability level = .481 | |  |  |  |  |  |  |  |  |
| --- | --- | --- | --- | --- | --- | --- | --- | --- | --- |
| Standardized Direct Effects | |  |  |  |  |  |  |  |  |
|  | Year | Precipitation | LCC | LNC | LDMC | SLA | LA | LT | height |
| LCC | 0 | 0.272 | 0 | 0 | 0 | 0 | 0 | 0 | 0 |
| LNC | -0.796 | 0 | 0 | 0 | 0 | 0 | 0 | 0 | 0 |
| LDMC | 0.669 | 0.202 | 0 | 0 | 0 | 0 | 0 | 0 | 0 |
| SLA | -0.804 | 0 | 0 | 0 | 0 | 0 | 0 | 0 | 0 |
| LA | -0.799 | 0 | 0 | 0 | 0 | 0 | 0 | 0 | 0 |
| LT | 0.663 | 0 | 0 | 0 | 0 | 0 | 0 | 0 | 0 |
| height | 0.345 | 0.226 | 0 | 0 | 0 | 0 | 0 | 0 | 0 |
| Aboveground biomass | 0.566 | 0.371 | -0.101 | 0.012 | -0.072 | -0.111 | 0.31 | -0.031 | 0.327 |
| Standardized Indirect Effects | | |  |  |  |  |  |  |  |
|  | Year | Precipitation | LCC | LNC | LDMC | SLA | LA | LT | height |
| LCC | 0 | 0 | 0 | 0 | 0 | 0 | 0 | 0 | 0 |
| LNC | 0 | 0 | 0 | 0 | 0 | 0 | 0 | 0 | 0 |
| LDMC | 0 | 0 | 0 | 0 | 0 | 0 | 0 | 0 | 0 |
| SLA | 0 | 0 | 0 | 0 | 0 | 0 | 0 | 0 | 0 |
| LA | 0 | 0 | 0 | 0 | 0 | 0 | 0 | 0 | 0 |
| LT | 0 | 0 | 0 | 0 | 0 | 0 | 0 | 0 | 0 |
| height | 0 | 0 | 0 | 0 | 0 | 0 | 0 | 0 | 0 |
| Aboveground biomass | -0.124 | 0.032 | 0 | 0 | 0 | 0 | 0 | 0 | 0 |
